# BAG2 drives chemoresistance of breast cancer by exacerbating mutant p53 aggregate

**DOI:** 10.7150/thno.78492

**Published:** 2023-01-01

**Authors:** Xinjian Huang, Dongni Shi, Xuxiazi Zou, Xuxia Wu, Shumei Huang, Lingzhi Kong, Muwen Yang, Yunyun Xiao, Boyu Chen, Xiangfu Chen, Ying Ouyang, Libing Song, Yunting Jian, Chuyong Lin

**Affiliations:** 1 Department of Experimental Research, State Key Laboratory of Oncology in South China, Collaborative Innovation Center for Cancer Medicine, Sun Yat-sen University Cancer Center, Guangzhou 510060, China.; 2 Department of Breast Surgery, Sun Yat-sen University Cancer Center, Guangzhou 510060, China.; 3 Department of Biochemistry, Zhongshan School of Medicine, Sun Yat-sen University, Guangzhou 510080, China.; 4 Key Laboratory of Protein Modification and Degradation, School of Basic Medical Sciences; Guangzhou Institute of Oncology, Tumor Hospital, Guangzhou Medical University, 511436, Guangzhou, China.; 5 Department of Pathology, Key Laboratory of Reproduction and Genetics of Guangdong Higher Education Institutes, Key Laboratory for Major Obstetric Diseases of Guangdong Province, The Third Affiliated Hospital of Guangzhou Medical University, Guangzhou 510150, China.

**Keywords:** breast cancer, chemoresistance, BAG2, mutant p53 aggregate, HSP90

## Abstract

**Rationale:** Chemoresistance is a major challenge in the clinical management of patients with breast cancer. Mutant p53 proteins tend to form aggregates that promote tumorigenesis in cancers. We here aimed to explore the mechanism for the generation of mutant p53 aggregates in breast cancer and assess its role in inducing chemoresistance.

**Methods:** Expression of BCL2-associated athanogene 2 (BAG2) was evaluated by qRT-PCR, western blotting, and immunohistochemistry in breast cancer patient specimens. The significance of BAG2 expression in prognosis was assessed by Kaplan-Meier survival analysis and the Cox regression model. The roles of BAG2 in facilitating the formation of mutant p53 aggregates were analyzed by co-immunoprecipitation, immunofluorescence, and semi-denaturing detergent-agarose gel electrophoresis assays. The effects of BAG2 on the chemoresistance of breast cancer were demonstrated by cell function assays and mice tumor models.

**Results:** In the present study, we found that BAG2 was significantly upregulated in relapse breast cancer patient tissues and high BAG2 was associated with a worse prognosis. BAG2 localized in mutant p53 aggregates and interacted with misfolded p53 mutants. BAG2 exacerbated the formation of the aggregates and recruited HSP90 to promote the propagation and maintenance of the aggregates. Consequently, BAG2-mediated mutant p53 aggregation inhibited the mitochondrial apoptosis pathway, leading to chemoresistance in breast cancer. Importantly, silencing of BAG2 or pharmacological targeting of HSP90 substantially reduced the aggregates and increased the sensitivity of chemotherapy in breast cancer.

**Conclusion:** These findings reveal a significant role of BAG2 in the chemoresistance of breast cancer via exacerbating mutant p53 aggregates and suggest that BAG2 may serve as a potential therapeutic target for breast cancer patients with drug resistance.

## Introduction

Breast cancer is the most common cancer and one of the leading causes of cancer-related death among women worldwide 1. It is a highly heterogeneous disease with chemotherapeutic intervention being an important regimen in the treatment of patients 2, 3. However, chemoresistance impedes efficacy, leading to tumor recurrence, metastasis, and patient death 4. Therefore, a better understanding of the potential mechanism of chemoresistance is vital for improving the treatment of breast cancer.

Accumulating evidence revealed that mutant p53 aggregates play an important role in tumorigenesis in several cancers 5-7. The ability to form mutant p53 aggregates depends on the mutated sites of p53 8. Over 95% of p53 mutations occur in the DNA-binding domain (DBD) and affect either the ability to bind DNA (contact mutants) or the structural stability (structural mutants) 9. Structural p53 mutants are prone to form misfolded proteins, that interact with many functional proteins to form co-aggregates distributed in the cytoplasm. Whether mutant p53 aggregates participate in breast cancer chemoresistance is unclear. A better understanding of the mechanism for the formation of mutant p53 aggregates and their roles in chemoresistance might provide clues for breast cancer therapy.

Chaperones and co-chaperones are associated with cellular protein quality by controlling protein to elongate or deal with misfolded and/or aggregated proteins 10-12. BCL2-associated athanogene 2 (BAG2) is a negative regulator of the carboxyl terminus of HSC70-interacting protein (CHIP), and serves as a mediator of the molecular chaperone HSP90/HSC70, which could abrogate the degradation of misfolded substrate proteins 12, 13. Unlike other BAG family proteins, BAG2 binds clients via its brand-new BAG (BNB) domain, an atypical dimeric C-terminal structure, and forms higher-order oligomers via its coiled-coil (CC) domain in the N-terminal domain (NTD) 14. BAG2 is associated with the formation of protein aggregates 10 that appear as puncta along with tau aggregates in Alzheimer's disease 15. In addition, BAG2 also interacts with PINK1, a Parkinson's disease-related protein, and directly binds and stabilizes both wild-type PINK1 and the R492X PINK1 mutant by decreasing their ubiquitination, which contributes to the pathogenesis of Parkinson's disease 16. Notably, upregulation of BAG2 is found in many types of cancer, including gastric cancer, glioma, hepatocellular carcinoma, oral cancer, and colorectal cancer, and is associated with the malignant progression of cancer 17-21. Recently, BAG2 was reported to enhance mutant p53 levels and accumulation, and promote tumorigenesis in tumors 22. Furthermore, BAG2 is upregulated in triple-negative breast cancer and is associated with poor patient survival 23. However, whether BAG2 promotes mutant p53 aggregation and confers chemoresistance in breast cancer remains unknown.

In the present study, we found that BAG2 was significantly upregulated in relapse breast cancer specimens and served as an independent predictor of poor prognosis in patients with breast cancer. BAG2 promotes aggregation of misfolded mutant p53 by directly binding to misfolded p53 mutants and recruiting HSP90 to ensure the propagation and maintenance of the aggregates. Importantly, knockdown of BAG2 or pharmacological targeting of HSP90 selectively abrogates the aggregates and renders breast cancer cells hypersensitive to chemotherapeutic drugs. These data revealed an important mechanism for breast cancer chemoresistance and suggested that BAG2 and mutant p53 aggregates may serve as promising targets against breast cancer.

## Materials and methods

### Cells

The human osteosarcoma cell line Saos2, breast cancer cell lines MCF-7, SK-BR-3, MDA-MB-468, and BT-549, and mouse breast cancer cell line 4T1 were obtained from ATCC (Manassas, VA, USA). All cells used in this study were authenticated using short tandem repeat (STR) profiling and propagated in the recommended serum-supplemented medium.

### Immunofluorescence (IF) Staining

Cells (5 × 10^4^ cells/well) were seeded onto coverslips. After 24 h, the cells were washed three times with phosphate-buffered saline (PBS) and fixed with 4% (v/v) paraformaldehyde for 15 min at room temperature. After rinsing with PBS, cells were permeabilized and blocked with 0.2% (v/v) Triton X-100 and 1% (w/v) bovine serum albumin (BSA) in PBS for 1 h. The cells were then stained with the following primary antibodies: anti-p53 (1:100, mouse DO-1 monoclonal, Biotechnology, Santa Cruz, CA, USA) and anti-BAG2 (1:50, rabbit polyclonal, Sigma-Aldrich, St Louis, MO, USA) for 2 h at 4 °C according to the manufacturer's instructions. The secondary antibodies (goat anti-mouse Alexa Fluor 488 or goat anti-rabbit Alexa Fluor 594, Cell Signaling Technology) were diluted 1:1000 and incubated for 1 h. After washing thrice with PBS, the cells were counterstained with DAPI (Sigma-Aldrich) to visualize the nuclei. Images were captured using an epifluorescence microscope. The number of cells with merged puncta was counted in five random view fields. This counting was repeated three times, and the values are shown as the mean ± S.D.

### Semi-denaturing detergent-agarose gel electrophoresis (SDD-AGE)

SDD-AGE was used to examine SDS-resistant aggregates, as previously reported 24. Briefly, cells were harvested by centrifugation and re-suspended in a lysis buffer (1 M Tris, [pH 7.4]; 5 M NaCl; 1% [v/v] glycerol, 1% [v/v] Triton X-100) containing 1 × Protease Inhibitor cocktail (Sigma-Aldrich). The protein concentrations of the cell lysates were adjusted and then mixed with 4 × sample buffer (10 × TAE, 20% [v/v] glycerol, 4% [v/v] SDS, bromophenol blue). The samples were incubated at room temperature for 15 min and loaded onto a 2.0% agarose gel containing 1 × TAE and 0.1% SDS. The gel was run at 60 V for approximately 4 h with TAE containing 0.1% SDS as the running buffer, and then transferred to a polyvinylidene difluoride (PVDF) membrane using the capillary transfer method. The PVDF membranes were then incubated with the indicated antibodies, and the bands were visualized using an ECL reagent.

### Immunoprecipitation (IP) assays

Lysates were prepared from the indicated cancer cells using a lysis buffer (150 mM NaCl, 10 mM HEPES, [pH 7.4], 1% NP-40), and the lysates were incubated with the anti-p53 (DO-1, PAb240, or PAb1620), anti-BAG2 antibodies with protein G agarose, Flag or HA affinity agarose (Sigma-Aldrich), overnight at 4 °C. Beads containing affinity-bound proteins were washed six times with the immunoprecipitation wash buffer (150 mM NaCl, 10 mM HEPES, [pH 7.4], and 0.1% NP-40), followed by elution with 1 M glycine [pH 3.0]. The eluates were then mixed with the sample buffer, denatured, and electrophoresed for western blotting analysis.

### Construction of plasmids and stable cell lines

Genes and their truncated forms with or without affinity-tagged bait proteins were cloned into the pLVX-IRES-Hyg vector (Clontech, Mountain View, CA, USA) for overexpression. Short hairpin RNA (shRNA) oligonucleotides targeting the mRNAs of endogenous genes were cloned into a pSuper-retro-neo vector to silence gene expression. The shRNA sequences targeting BAG2 were as follows: shRNA#1, 5'-GUGGUCAAUAAGUUUCUGGAU-3'; shRNA#2, 5'-GAUCAGAAGUUUCAAUCCAUA-3'. Mutations in clones were created using a Stratagene mutagenesis kit according to the manufacturer's protocol. All the clones used in this study were validated by sequencing. Plasmids were transduced into cells using Lipofectamine 3000 reagent (Invitrogen, Carlsbad, CA, USA) according to the manufacturer's instructions. Stable cells were generated from cell pools by retroviral infection of HEK293T cells and selected with 50 μg/mL hygromycin B or 250 μg/mL G418 (Geneticin) for 10 days. The transduction of genes or shRNAs was further validated by western blotting analysis. The BAG2-knockout Saos2 (Saos2-BAG2^-/-^) cell line was generated using the CRISPR/Cas9 system (24157548). Briefly, a small guide RNA (sgRNA) was designed to target the first exon of BAG2 and induce cleavage by Cas9. A single-stranded DNA oligonucleotide (ssODN) with 40-nt homologous nucleotides on either side of the cleaved region and an insertion of multiple stop codon sequence “TAGATAACTGAT” was used as the repair donor for homology-directed repair.

### Patient specimens

This study was conducted on 236 paraffin-embedded breast cancer samples (collected between 2002 and 2013) that were histopathologically and clinically diagnosed at the Sun Yat-sen University Cancer Center. The clinicopathological characteristics of the patient are summarized in Table S1. Samples were obtained from patients who had received chemotherapy for the disease. The samples were staged according to the American Joint Committee on Cancer 7^th^ edition staging system. Ethical approval and patient consent were obtained from the Institutional Research Ethics Committee for the use of clinical specimens for research purposes. Tumor samples with ≥ 60% tumor nuclei and ≤ 20% necrosis were analyzed for protein expression examination analysis.

### Immunochemistry (IHC) staining

In the present study, IHC staining was performed on 236 human breast cancer specimens to determine the expression levels of misfolded p53 and BAG2. Briefly, paraffin-embedded specimens were cut into 4 μm sections and baked at 65 °C for 30 min. Sections were deparaffinized with xylene, rehydrated, submerged in EDTA antigen retrieval buffer, and microwaved for antigen retrieval. The sections were then treated with 3% hydrogen peroxide in methanol to quench endogenous peroxidase activity. This was followed by incubation with 1% BSA to block nonspecific binding, and then incubation with primary antibodies overnight at 4 °C. After washing, the tissue sections were treated with biotinylated anti-rabbit secondary antibody, followed by further incubation with streptavidin-conjugated horseradish peroxidase (HRP) complex (Zsbio, Beijing, China). Finally, the sections were immersed in 3-amino-9-ethyl carbazole, counterstained with 10% Mayer's hematoxylin, dehydrated, and mounted in crystal mount. The primary antibodies used in immunohistochemical staining included anti-p53 (mouse PAb240 monoclonal, BD Biosciences, San Jose, CA, USA, 1:50), and anti-BAG2 (rabbit polyclonal, Sigma-Aldrich, 1:100). The specificity of the antibodies was validated using IHC staining. Staining results were evaluated and scored by two independent pathologists, who were blinded to the clinical outcomes. Tumor cell proportions were scored as follows: 0: no positive tumor cells; 1, < 10%; 2, 10%-35%; 3, 35%-75%; 4, > 75%. Staining intensity was graded according to the following standard: 0, no staining; 1, weak staining (light yellow); 2, moderate staining (yellow brown); 3, strong staining (brown). The staining index (SI) was calculated as the proportion of positive cells and staining intensity score. Using this assessment method, we evaluated protein expression in breast cancer tissues by measuring the SI, with possible scores of 0, 1, 2, 3, 4, 6, 8, 9, and 12. The cut-off values to define high and low BAG2 expression were derived from the highest combined sensitivity and specificity for patient survival. An optimal threshold of SI ≥ 6 was then determined to define samples with high BAG2 expression and samples with SI < 6 were determined to have low expression.

### Xenograft models

Female BALB/c-nu mice (5-6 weeks old, 18-20 g) were purchased and housed in barrier facilities on a 12-h light/dark cycle. The Institutional Animal Care and Use Committee of Sun Yat-sen University approved all experimental procedures. To establish the orthotopic xenograft model, 2 × 10^6^ SK-BR-3 cells with or without BAG2 knockdown were injected into the fat pads or dorsal flanks of the mice. One week after inoculation, mice were intraperitoneally injected with paclitaxel (PTX, 10 mg/kg, twice a week) for four weeks. The tumor volumes were determined every week. The tumor volume was calculated using the following equation: (L × W^2^)/2. The mice were sacrificed, and the tumors were isolated and weighed. Serial 6.0-μm sections were cut and stained with anti-BAG2, anti-Ki67, and terminal deoxynucleotidyl transferase nick-end-labeling (TUNEL) staining, to determine the level of apoptosis.

For spontaneous metastasis assays, cells of the mouse breast cancer cell line 4T1-luciferase (1 × 10^5^) were orthotopically injected into the mammary fat pads of mice and incubated for one month. Metastasis was detected using an *in vivo* imaging system (IVIS; Caliper) by blocking the signal from primary xenografts. Mice were euthanized one month later, and their lungs were fixed in formalin and embedded in paraffin using a routine method. Lung surface metastatic lesions were counted under a dissecting microscope and presented as the mean ± S.D. Lung metastasis was assessed using hematoxylin and eosin (H&E) staining.

The HSP90 inhibitor IPI-504 (100 mg/kg, twice a week) was administered intraperitoneally one week after the tumor inoculation. Tumor volumes were determined weekly and the number of lung metastases was counted to determine the efficacy of IPI-504 in terms of its effect on tumor growth and metastasis. Tumor sections were subjected to IHC and TUNEL staining.

### Western blotting analysis

Total proteins were extracted from both tissue and cell samples using radioimmunoprecipitation assay (RIPA) lysis buffer supplemented with a protease/Phosphatase inhibitor cocktail (Cell Signaling Technology). The extracted protein was quantified using bicinchoninic acid assay (Pierce, Rockford, IL, USA) according to the manufacturer's instructions. Samples containing the same amount of protein were separated using SDS-PAGE and transferred to PVDF membranes. The membranes were blocked with 5% skim milk in (Tris-buffered saline with 0.1% Tween-20) for 1 h, and subsequently incubated with the appropriate primary antibodies overnight at 4 °C. After washing thrice in TBST, the membranes were incubated with HRP-conjugated secondary antibodies for 1 h at room temperature. The following antibodies were used: anti-p53 (mouse DO-1 monoclonal, Santa Cruz, 1:1000), anti-BAG2 (rabbit polyclonal, Sigma-Aldrich, 1:1000), anti-Bax (rabbit polyclonal, Abcam, 1:1000), anti-PINK1 (rabbit polyclonal, CST, 1:1000), anti-VDAC (rabbit polyclonal, CST, 1:1000), anti-cytochrome c (rabbit polyclonal, CST, 1:1000) and anti-α-tubulin (mouse monoclonal, Sigma-Aldrich, 1:4000).

### Chemical reagents

The HSP90 inhibitor IPI-504 was purchased from AdooQ (Irvine, CA, USA), and was used at a concentration of 2.5 μM *in vitro* or 100 mg/kg *in vivo*. The proteasome inhibitor MG132 was purchased from Selleck Chemicals (Houston, TX, USA) and was used at a concentration of 10 μM.

### Statistical analysis

All statistical analyses were performed using the SPSS version 19.0 statistical software package (IBM Corp., Armonk, NY, USA). The statistical tests used for data analysis included the Student's t-test (two-tailed), χ^2^ test (two-tailed), and log-rank test. Multivariate statistical analysis was performed using the Cox regression model. Statistical significance was set at *P <* 0.05.

## Results

### High BAG2 expression correlates with chemoresistance and poor prognosis in breast cancer

**Figure 1:**
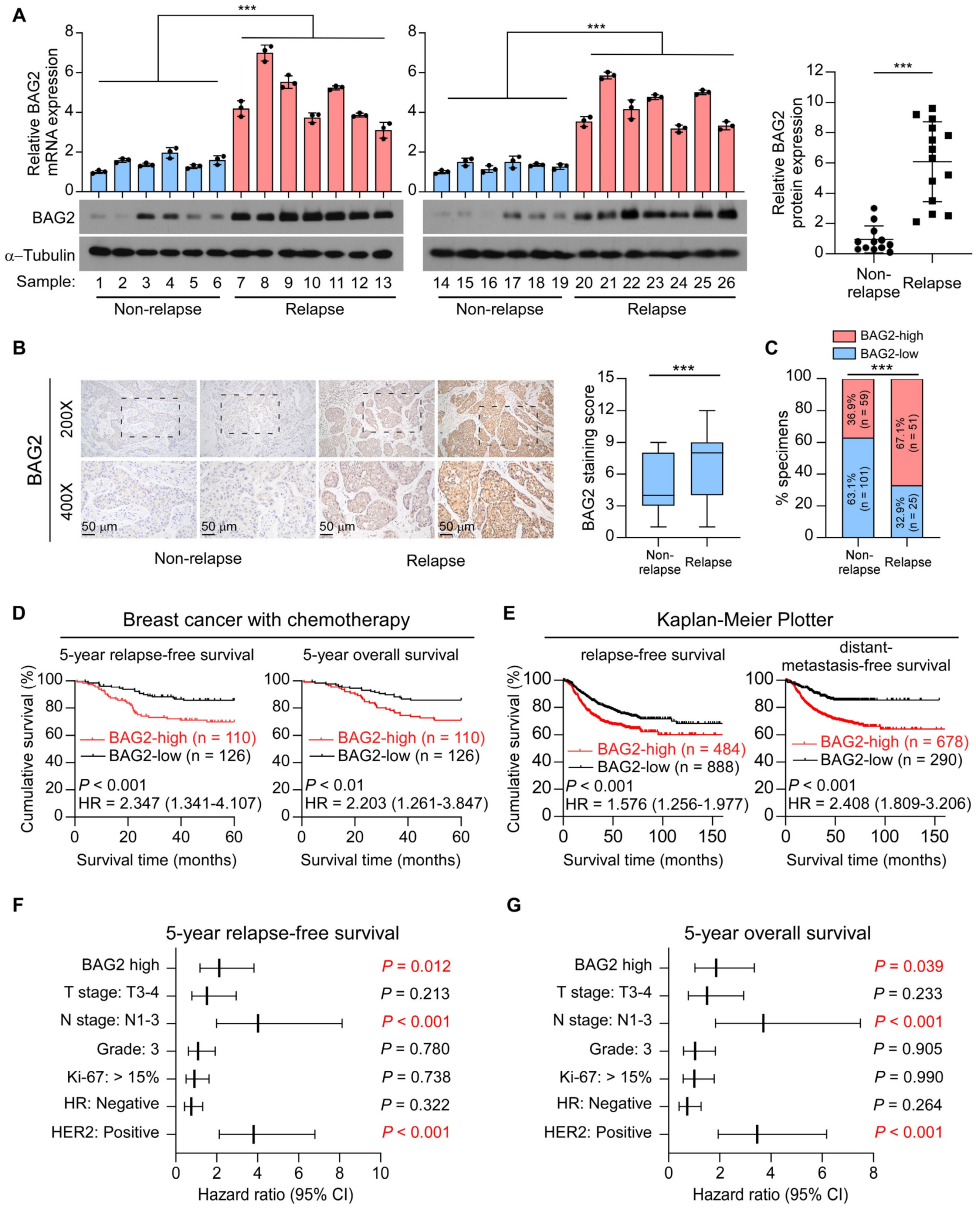
** High BAG2 expression correlates with chemotherapy resistance and poor prognosis of breast cancer. (A)** Left: The mRNA (upper) and protein (lower) expression levels of BAG2 in breast cancer tissues, including 12 non-relapse samples and 14 relapse samples. The mRNA and protein expression were both normalized to GAPDH. Right: Statistic analysis of BAG2 expression density in different groups.** (B)** Representative images (left) and staining index (right) of BAG2 expression in non-relapse (n = 160) and relapse (n = 76) breast cancer tissues under treatment of chemotherapy. Two-sided Student's t-test was used for statistical analysis. **(C)** Correlation analysis between BAG2 expression and relapse status in patients. Two-sided χ^2^ test was used to evaluate the correlation.** (D)** Kaplan-Meier curve of 5-year RFS (left) and 5-year OS (right) for breast cancer patients treated with chemotherapy with high expression of BAG2 (BAG2-high, n = 110) versus those with low expression of BAG2 (BAG2-low, n = 126). Log-rank test was used for statistical analysis. **(E)** Kaplan-Meier curve of RFS (left) and DMFS (right) for breast cancer patients treated with chemotherapy with high expression of BAG2 versus those with low expression of BAG2 from the online database (http://kmplot.com/analysis). Log-rank test was used for statistical analysis.** (F-G)** Multivariate Cox regression analysis evaluates the significance of the association between BAG2 expression and 5-year RFS and 5-year OS in the presence of other clinical variables. Error bar in A represents the mean ± SD of three biological replicates. Two-sided Student's t test was used for statistical analysis. ***P < 0.001.

Since chemoresistance is associated with rapid recurrence, we first examined BAG2 expression in 26 primary tumor tissues, including 12 non-relapse tissues and 14 relapse tissues. Remarkably, both mRNA and protein expression levels of BAG2 were significantly upregulated in relapse tissues compared to non-relapse tissues (Figure 1A). We then performed immunohistochemistry (IHC) to analyze BAG2 expression in a cohort of 236 patients with breast cancer who received chemotherapy (Table S1). Consistently, IHC staining revealed that BAG2 was significantly overexpressed in relapse specimens compared to non-relapse specimens in breast cancer (Figure 1B-C). Moreover, Kaplan-Meier curve analysis and log-rank test revealed that patients with high expression of BAG2 had shorter 5-year relapse-free survival (hazard ratio (95% CI) = 2.347 (1.341-4.107); *P* < 0.001) and 5-year overall survival (hazard ratio (95% CI) = 2.203 (1.261-3.847); *P* < 0.01) compared to patients with low-BAG2 level (Figure 1D). The prognostic value of BAG2 was also assessed using the public program Kaplan-Meier Plotter (http://kmplot.com/analysis). The Kaplan-Meier Plotter analysis showed that high expression of BAG2 was significantly associated with poor relapse-free survival (hazard ratio (95% CI) = 1.576 (1.256-1.977); *P* < 0.001) and distant-metastasis-free survival (hazard ratio (95% CI) = 2.408 (1.809-3.206); *P* < 0.001) in breast patients with chemotherapy (Figure 1E). Interestingly, the analysis showed that BAG2 expression was not associated with tumor relapse in all HER2-positive breast cancer patients; however, when only those with chemotherapy were tested, it showed that high BAG2 expression was significantly associated with tumor relapse, indicating that BAG2 may be involved in the tolerance to chemotherapy, instead of HER2-targeting therapy (Figure S1A-B). BAG2 expression was associated with tumor relapse status, but not correlated with HER2 status or lymph node metastasis (Table S2). Notably, multivariate Cox regression analysis indicated that when multiple variables were taken into account, variables including BAG2 expression-high, lymph node metastasis-positive, and HER2 status-positive were independent prognostic markers that could significantly predict poor outcomes in these patients (Figure 1F-G). Taken together, these results suggest that BAG2 may play a role in the progression of breast cancer with chemotherapy.

### BAG2 contributes to chemoresistance in breast cancer

**Figure 2:**
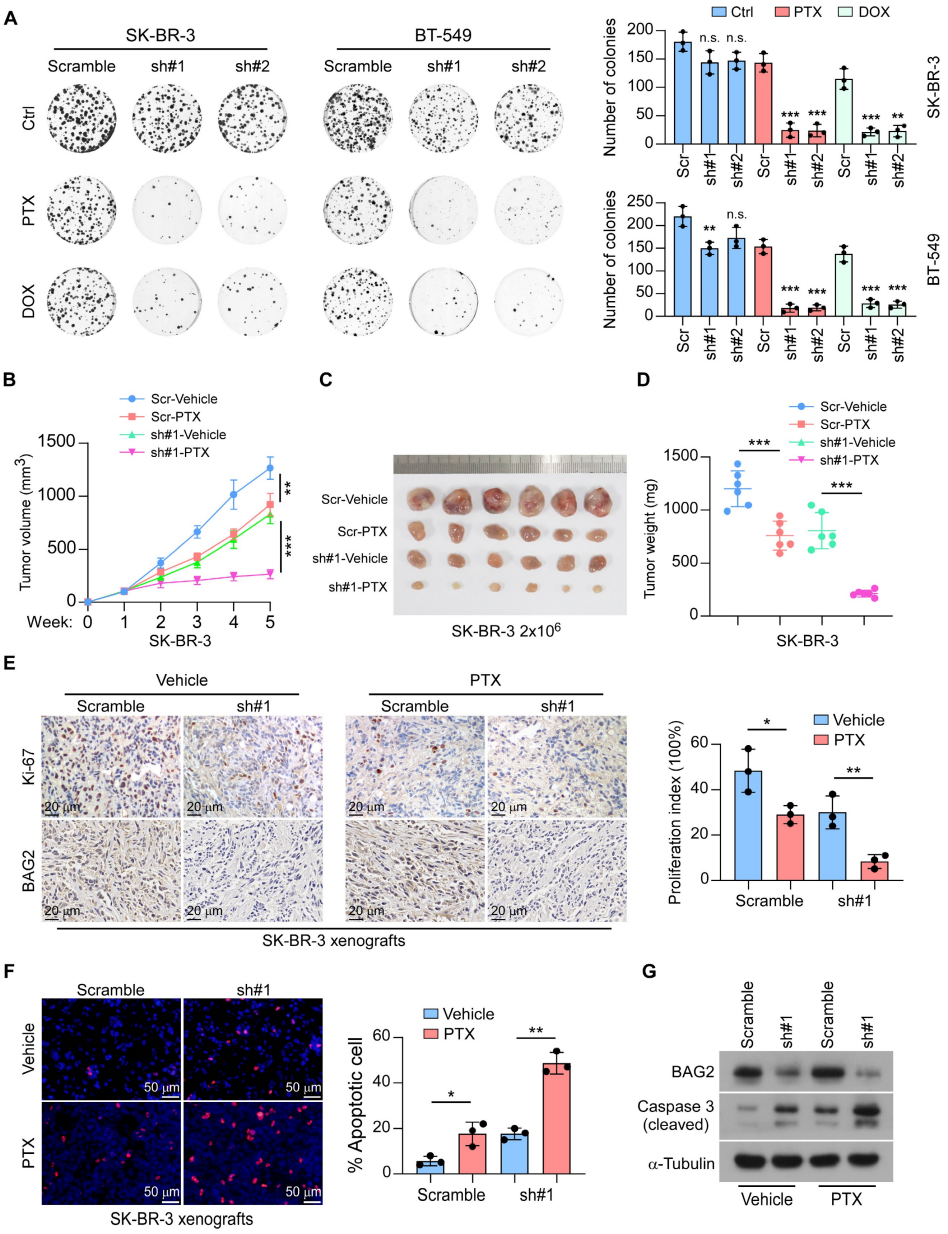
** BAG2 contributes to chemoresistance in breast cancer. (A)** Representative images (left) and quantification (right) of colony formation formed by the indicated cells (SK-BR-3 and BT-549) treated with or without PTX or DOX. **(B-D)** Orthotopic tumor model of the indicated cells (SK-BR-3 and SK-BR-3-sh#1) with or without PTX treatment. B: tumor volume of mice generated by the indicated cells; C: Representative images of the orthotopic tumors generated by SK-BR-3 cells; D: tumor volume of mice generated by the indicated cells.** (E)** Representative Ki-67 staining images and statistic quantification of proliferation index in the indicated tumors. **(F)** Representative images and statistic quantification of TUNEL assays. **(G)** BAG2 and cleaved Caspase 3 expression were detected by western blotting in the indicated tumors with or without silencing BAG2, under PTX treatment. Each error bar in A, E, and F represents the mean ± SD of three biological replicates. Each error bar in B and D represents the mean ± SD derived from tumor mouse models (n = 6 mice/group). Two-sided Student's t-test (A, E, F), or One-way repeated-measures ANOVA test (B and D) was used for statistical analysis. *P < 0.05, **P < 0.01, ***P < 0.001, and n.s.: not significant.

We next explored the role of BAG2 in the chemoresistance of breast cancer. Strikingly, the colony surviving assays showed that knockdown of BAG2 increased the sensitivity of SK-BR-3 and BT-549 cell lines to treatment with paclitaxel (PTX) and doxorubicin (DOX) (Figure 2A). The effects of BAG2 in chemotherapy were further tested using the SK-BR-3 xenograft model. Scramble or BAG2-silencing SK-BR-3 cells were orthotopically injected into the fat pads of mice, and tumor burden was measured regularly. One week later, mice were given intraperitoneal injections of vehicle or 10 mg/kg PTX (twice a week). As shown in Figure 2B-D, the depletion of BAG2 facilitated the efficacy of paclitaxel (PTX) in suppressing SK-BR-3 tumor growth. Analysis of the SK-BR-3 xenografts revealed that co-administration of BAG2 knockdown and PTX treatment robustly decreased the proliferation index but promoted cell apoptosis in the SK-BR-3 tumors (Figure 2E-G). These findings reveal that BAG2 contributes to chemoresistance in breast cancer.

### BAG2 promotes mutant p53 aggregates formation

**Figure 3:**
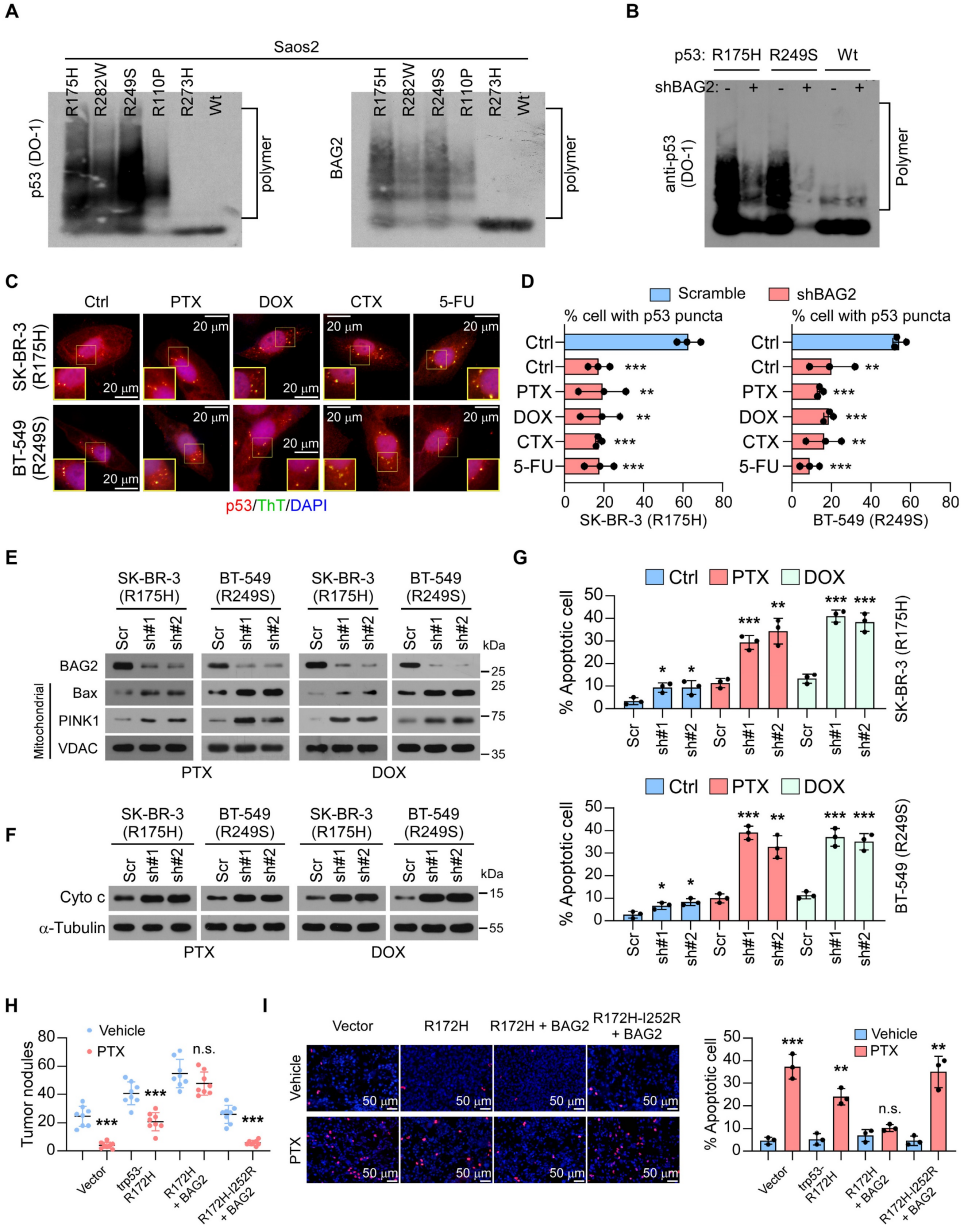
** BAG2 promotes mutant p53 aggregates and increases the anti-apoptosis ability of breast cancer cells. (A)** Semi-denaturing detergent agarose gel electrophoresis (SDD-AGE) assays examined the SDS-resistant amyloid-like fibers formed by p53 and BAG2 polymers in the indicated cell lysates. **(B)** SDD-AGE assays indicate the SDS-resistant amyloid-like fibers formed by p53 in Saos2 cells expressing p53-R175H, -R249S, and wild-type, with or without silencing BAG2. **(C)** SK-BR-3 (R175H) and BT-549 (R249S) cells under stationary phase (Ctrl), PTX, DOX, CTX (Cyclophosphamide), and 5-FU (5-Fluorouracil) treatment were subjected to immunofluorescence (IF) staining of p53 (red) with anti-p53 DO-1 and Thioflavin T (green). Cell nuclei were counterstained with DAPI (blue). Quantification was shown as the % of the total number of cells ± S.D. by counting the number of cells with p53 puncta from three to five different fields. **(D)** SK-BR-3 (R175H) and BT-549 (R249S) cells, with or without BAG2 knockdown were analyzed using IF staining to examine the p53 aggregated puncta. **(E)** Mitochondrial fractions were isolated, and BAG2, Bax, and PINK1 expression were detected by western blotting in the indicated cancer cell lines with or without silencing BAG2, under PTX or DOX treatment; VDAC was used as a mitochondrial marker. **(F)** Western blotting analysis of the levels of cytochrome c (Cyto c) in the indicated cancer cell lines with or without silencing BAG2, under PTX or DOX treatment; α-Tubulin was used as a loading control.** (G)** Annexin V/PI assays of the indicated cells with or without PTX or DOX treatment.** (H)** Visible surface metastatic lesions in the indicated groups were counted. **(I)** TUNEL staining and the quantifications in the orthotopic tumors of 4T1 cells with or without PTX treatment. Each error bar in D, G and I represents the mean ± SD of three biological replicates. Each error bar in H represents the mean ± SD derived from tumor mouse models (n = 8 mice/group). Two-sided Student's t-test (D, G, H, I) was used for statistical analysis. *P < 0.05, **P < 0.01, ***P < 0.001; n.s.: not significant.

The status and expression of p53 mutants were associated with chemoresistance and a poor prognosis in human cancers by inhibiting cell apoptosis and decreasing sensitivity to anticancer drugs 25-28. Given that BAG2 was reported to interact with mutant p53 and mutant p53 aggregates were found to promote chemoresistance in ovarian cancer 29, we hypothesized that BAG2 might regulate the generation of mutant p53 aggregates to induce chemoresistance. Aggregates are amyloid-like fibers comprising SDS-resistant mega Dalton protein complexes 30. We transfected p53 structural mutants (tends to form aggregates) R175H, R282W, R249S, and R110P, and p53 contact mutants (does not form aggregates) R273H and wild-type (WT) p53 in p53-null Saos2 cells, and evaluated the formation of mutant p53 aggregates by semi-denaturing detergent agarose gel electrophoresis (SDD-AGE) assays. As expected, SDD-AGE assays showed that mutant p53 aggregates were formed in Saos2 cells with p53 structural mutants (R175H, R282W, R249S, and R110P) but not in cells with p53 contact mutant (R273H) or WT p53 (Figure 3A). Notably, we observed a positive correlation between BAG2 expression and the formation of mutant p53 aggregates indicating a potential role of BAG2 in the aggregates (Figure 3A). Since p53-R175H has the highest occurrence (5.98%) in breast cancer and p53-R249S also has high-level of amyloid-like p53 aggregates, these two mutants were used to investigate the role of BAG2 in the formation of mutant p53 aggregates. Notably, silencing of BAG2 robustly decreased the levels of amyloid-like p53 aggregates in Saos2-R175H and Saos2-R249S cells but not in Saos2 cells with WT p53 (Figure 3B). Moreover, we performed immunofluorescence to measure perinuclear puncta as the structural mutant p53 proteins tend to aggregate in perinuclear puncta. We found that treatment with chemotherapeutic drugs increased p53 perinuclear puncta in SK-BR-3 and BT-549 cells, which had the R175H mutation and R249S in *TP53* respectively, but no obvious puncta were found in MDA-MB-468 and MCF-7 cells harboring the R273H mutation in *TP53* and wild-type *TP53* respectively (Figure 3C and Figure S2A-B). Importantly, knockdown of BAG2 significantly decreased p53 perinuclear puncta in SK-BR-3 (R175H) and BT-549 (R249S) cells with or without chemotherapeutic drug treatments (Figure 3D). These results indicate that BAG2 plays a role in the production of mutant p53 aggregates.

### BAG2-induced mutant p53 aggregates endow breast cancer cells with resistance to apoptosis

We further explored the oncogenic functions of the BAG2-induced mutant p53 aggregates. It is well established that p53 tightly regulates cell growth by promoting apoptosis and serves as a tumor suppressor 31, 32. As expected, we found that the knockdown of BAG2 in SK-BR-3 (R175H) and BT-549 (R249S) cells promoted the translocation of apoptotic enablers, Bax and PINK1 which induced outer mitochondrial membrane permeabilization, to the mitochondria, leading to cytochrome c (Cyto c) release (Figure 3E-F) with PTX or DOX treatment. Downregulation of BAG2 significantly enhanced apoptosis in SK-BR-3 (R175H) and BT-549 (R249S) cells treated with PTX and DOX, as indicated by annexin V/PI assays (Figure 3G). However, BAG2 depletion did not affect the sensitivity of MDA-MB-468 (R273H) and MCF-7 (WT) cells to chemotherapeutic drugs (Figure S2C).

Moreover, we constructed a mouse model of breast cancer by using a p53-null mouse 4T1 breast cancer cell line. We found that BAG2 promoted the effects of trp53-R172H (equivalent to the human R175H mutation) on tumor growth and anti-apoptosis with or without PTX treatment (Figure 3H-I). Importantly, the introduction of 4T1-BAG2-trp53^R172H-I252R^, which is equivalent to the human I254R mutation (the p53 mutation I254R is an aggregate-suppressing mutation 33), inhibited the effects of BAG2 on tumor growth and apoptosis following treatment with PTX (Figure 3H-I). These results indicate that BAG2 promotes breast cancer chemoresistance by inhibiting cell apoptosis via mutant p53 aggregates.

### Roles of BAG2 BNB and NTD structures in mutant p53 aggregates formation

**Figure 4:**
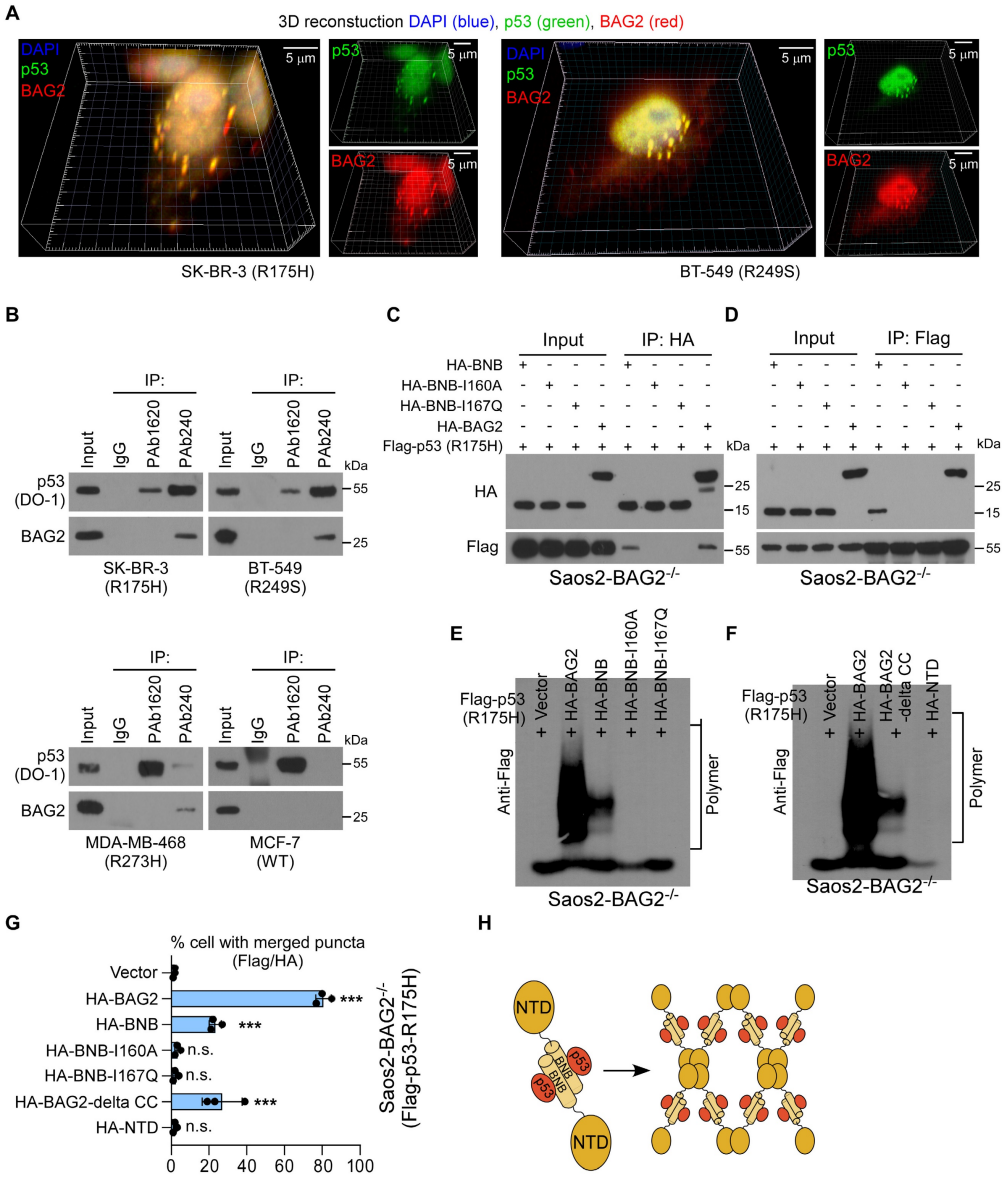
** Roles of BAG2 BNB and NTD structures in mutant p53 aggregates formation. (A)** Three-dimensional (3D) confocal microscopy revealed marked p53/BAG2 co-aggregates throughout the perinucleus.** (B)** Immuno-precipitation (IP) of native and misfolded p53 proteins with PAb1620 and PAb240 antibodies, followed by western blotting analysis to examine the interaction with BAG2. **(C)** Saos2-BAG2^-/-^ cells were transfected with the indicated HA-tagged BAG2 truncated forms, followed by IP assays to examine their interaction with p53. **(D)** IP assays showing the interactions between p53-R175H and the BAG2-BNB domain or the BNB-I160A and BNB-I167Q mutants in Saos2-BAG2^-/-^ cells. **(E)** SDD-AGE assays showing the amyloid-like fibers of the p53-R175H protein in Saos2-BAG2^-/-^ cells transfected with wild-type BAG2, and BAG2 with the BNB I160A and I167Q mutations.** (F)** Amyloid-like fibers of the p53-R175H protein in Saos2-BAG2^-/-^ cells transfected with delta-CC, NTD, and full-length BAG2. **(G)** Quantification of aggregates of p53 and BAG2 in Saos2-BAG2^-/-^ cells transfected with the indicated truncated forms of BAG2. **(H)** Schematic illustration of the BNB and NTD structures and the antiparallel dimer structure of the BAG2 protein. Error bar in G represents the mean ± SD of three biological replicates. Two-sided Student's t-test was used for statistical analysis. ***P < 0.001; n.s.: not significant.

The mechanism for BAG2-mediated aggregation of mutant p53 proteins was then investigated. 3D reconstruction analysis of immunofluorescence staining revealed that BAG2 was co-localized in R175H and R249S mutant p53 aggregates (Figure 4A). Aggregates are propagated by misfolded proteins 30. Therefore, we examined whether BAG2 interacts with misfolded p53 proteins in aggregates of p53 mutants. Two anti-p53 monoclonal antibodies were used, PAb240 and PAb1620, which specifically recognize misfolded and native p53, respectively 5, 34. Strikingly, interactions between p53 and BAG2 were observed by pull-down with PAb240, but not with PAb1620, suggesting that BAG2 selectively interacts with misfolded p53 (Figure 4B). The BNB domain of BAG2 enables it to form antiparallel dimers, tetramers, and even higher-order oligomers (Figure S3A) 14. To understand the details of the interactions between BAG2 and p53, truncated forms of BAG2 were expressed in a CRISPR/Cas-derived BAG2-knockout Saos2 (Saos2-BAG2^-/-^) cell line (Figure S3B-C). Co-immunoprecipitation (Co-IP) using an anti-HA antibody revealed that only truncated BAG2 with the BNB domain could bind to mutant p53-R175H, suggesting that the BNB domain is required for BAG2-mediated mutant p53 aggregates (Figure 4C). By forming an antiparallel dimer, BAG2 can simultaneously bind two substrates, whereas mutations at I160A and I167Q block the interaction with substrates 14. Consistent with previous reports, BNB mutations at I160A and I167Q abrogated their interactions with p53 (Figure 4C). Reciprocally, IP assays using antibodies against Flag-p53 confirmed the interaction with BNB in Saos2-BAG2^-/-^ cells (Figure 4D).

Furthermore, we performed SDD-AGE to examine the role of the BAG2-BNB domain in amyloid-like aggregates formations. Saos2-BAG2^-/-^ cells stably expressing mutant p53 (R175H) were transfected with various truncated forms of BAG2. BNB I160A and I167Q mutations, which lacked substrate-binding abilities abrogated the formation of amyloid-like aggregates (Figure 4E). Surprisingly, overexpression of the BNB domain resulted in only a slight increase in the levels of SDS-resistant amyloid-like fibers of the p53-R175H protein, and the fibers were smaller than those formed by full-length BAG2 (Figure 4E). Notably, the CC motif in the NTD of BAG2 is essential for its higher-order oligomerization of BAG2 14. Intriguingly, an SDD-AGE assay using cells expressing truncated BAG2 with restrained deletion of the CC motif showed a similar role to the BNB domain in the formation of amyloid-like fibers of the p53-R175H protein (Figure 4F). In contrast, no aggregates were formed after transfection with the NTD-containing fragment of BAG2 (Figure 4F). In addition, overexpression of BNB or delta CC-BAG2 in Saos2-BAG2^-/-^ cells had a limited ability to promote aggregation, generating four-fold fewer Flag/HA aggregates than full-length BAG2 (Figure 4G). BNB domain mutations completely abolished the aggregates, while transfection with the NTD fragment did not affect Flag/HA aggregates (Figure 4E-H and Figure S3D). These data indicate that both BNB and CC motifs are necessary, but not sufficient, for the complete function of BAG2 in the formation of amyloid-like aggregates.

### HSP90 is required for the propagation and maintenance of BAG2-mediated mutant p53 aggregates

**Figure 5:**
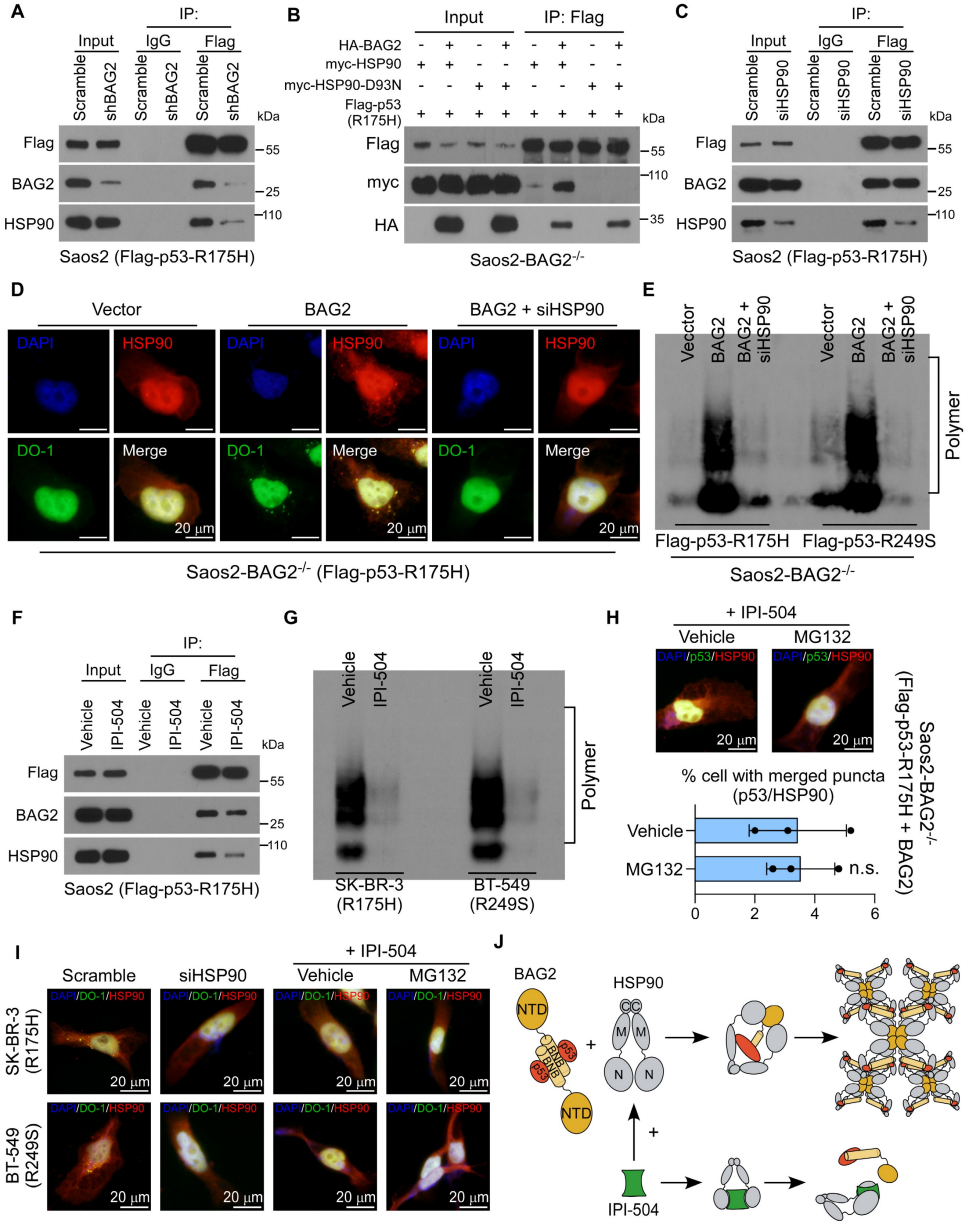
** HSP90 is required for the propagation and maintenance of BAG2-mediated mutant p53 aggregates. (A)** Saos2-p53-R175H cells were transfected with Scramble or BAG2 shRNA and followed by IP assays and analyzed by western blotting. **(B)** Saos2-BAG2^-/-^ cells were transfected with HA-BAG2, myc-HSP90 or myc-HSP90 D93N, and Flag-p53-R175H, and followed by IP assays and analyzed by western blotting. **(C)** Saos2-p53-R175H cells were transfected HSP90 siRNA and followed by IP assays and analyzed by western blotting. **(D)** Representative images of p53-R175H and HSP90 aggregates in Saos2-BAG2^-/-^ (Flag-p53-R175H) cells transfected with vector, BAG2, or BAG2 and HSP90 siRNA. **(E)** SDD-AGE assays indicate the amyloid-like fibers of p53-R175H and p53-R249S proteins in the lysates of Saos2-BAG2^-/-^ cells with the indicated transfections.** (F)** Saos2-p53-R175H cells were treated with the HSP90 inhibitor IPI-504 (2.5 μM, 48h) and followed by IP assays and analyzed by western blotting. **(G)** SDD-AGE assays indicate the amyloid-like fibers of endogenous p53 mutants in SK-BR-3 (R175H) and BT-549 (R249S) cells with or without IPI-504 treatment.** (H)** Representative images and quantification of p53/HSP90 aggregates in Saos2-BAG2^-/-^ cells transfected p53-R175H and BAG2 treating with or without IPI-504 and MG-132 (10 μM for 6 hours).** (I)** Representative images of p53/HSP90 aggregates in SK-BR-3 (R175H) and BT-549 (R249S) cells with the indicated treatments.** (J)** Schematic illustration of the interaction between BAG2 and HSP90 with or without treating IPI-504. Error bar in H represents the mean ± SD of three biological replicates. Two-sided Student's t-test was used for statistical analysis. n.s.: not significant.

Chaperones are engaged in protein aggregates, facilitating the propagation and maintenance of aggregates 35-37. We next investigated whether some other chaperone was required for BAG2-mediated mutant p53 aggregation. The chaperones HSP90 and HSP70, which were intensely associated with the conformation of p53 proteins 37-39, were then tested. Interestingly, co-IP assays indicated that knockdown of BAG2 in Saos2 cell expressing p53-R175H substantially reduced the interaction between HSP90 and p53-R175H whereas the HSP70-mutant p53 interaction remained unchanged (Figure 5A and Figure S4A). Re-expression of BAG2 rescued the interaction between HSP90 and p53-R175H in Saos2-BAG2^-/-^ cells (Figure 5B). In addition, an HSP90-D93N mutant, which is defective in ATP binding 40, could not interact with mutant p53-R175H and did not affect BAG2-p53-R175H binding (Figure 5B). Likewise, silencing of HSP90 did not alter the interaction between BAG2 and mutant p53-R175H (Figure 5C), indicating that the BAG2 and mutant p53-R175H interaction was independent of HSP90.

Notably, silencing of HSP90 dramatically repressed BAG2-induced aggregation of p53 mutants (R175H and R249S), as shown by immunofluorescence staining and SDD-AGE assays (Figure 5D-E and Figure S4B-D). Moreover, the HSP90 inhibitor, retaspimycin hydrochloride (IPI-504) 41, attenuated the binding between HSP90 and mutant p53-R175H, but did not affect the BAG2-mutant p53-R175H interaction (Figure 5F). SDD-AGE indicated that IPI-504 decreased mutant p53 aggregates (Figure 5G). Thus, these findings reveal that downregulation or inhibition of HSP90 abrogated BAG2-induced production of mutant p53 aggregates in both exogenously transfected and endogenous cell models, suggesting that HSP90 was required for the propagation and maintenance of aggregates.

To exclude the possibility that the suppressive effect of IPI-504 may result from increased p53 degradation rather than the suppression of mutant p53 aggregate propensity, we also measured the effect of IPI-504 on the formation of p53-R175H aggregates in the presence of the proteasome inhibitor MG-132. MG132 treatment did not rescue mutant p53 aggregates in the presence of IPI-504, suggesting that the role of HSP90 in mutant p53 aggregation was independent of its function in regulating p53 stability (Figure 5H-J and Figure S4E).

### Targeting BAG2-induced p53 aggregates by IPI-504 increases the sensitivity of chemotherapy

**Figure 6:**
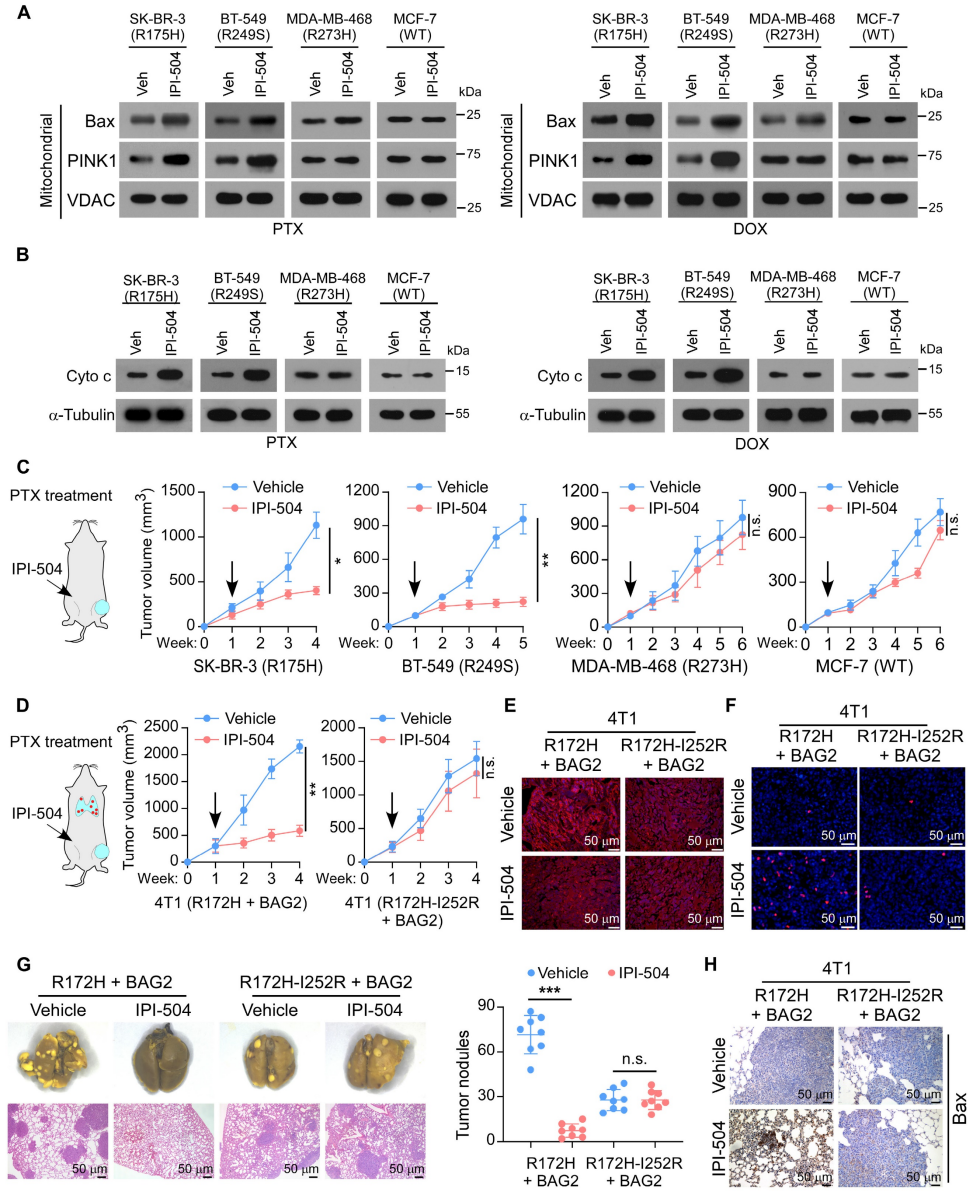
** Targeting BAG2-induced p53 aggregates by IPI-504 increases the sensitivity of chemotherapy. (A)** Mitochondrial fractions were isolated, and Bax and PINK1 expression were detected by western blotting in the indicated cancer cell lines with or without IPI-504, under PTX or DOX treatment; VDAC was used as a mitochondrial marker.** (B)** Western blotting analysis of the levels of Cyto c in the indicated cancer cell lines with or without IPI-504, under PTX or DOX treatment; α-Tubulin was used as a loading control. **(C)** Orthotopic xenograft model and tumor volumes of indicated orthotopic xenografts. One week after tumor inoculation, the mice were intraperitoneally injected with IPI-504 (100 mg/kg, twice a week) or vehicle, with co-administration of PTX.** (D)** Spontaneous metastasis model and tumor volumes of 4T1-BAG2-trp53R^172H^ and 4T1-BAG2-trp53R^172H-I252R^ orthotopic xenografts.** (E)** Aggregated p53 in the indicated sections xenografts were examined by IF staining. **(F)** TUNEL staining in the indicated orthotopic 4T1 tumors. **(G)** Images of lung metastases and quantification of visible surface metastatic lesions of the indicated 4T1 tumors.** (H)** IHC of Bax staining in the lung metastases. Each error bar in C, D, and G represents the mean ± SD derived from tumor mouse models (n = 8 mice/group). One-way repeated-measures ANOVA test (C, D) or Two-sided Student's t-test (G) was used for statistical analysis. ***P < 0.001, n.s.: not significant.

We further evaluated the therapeutic potential of IPI-504 in breast cancers that expressed mutant p53 aggregates. The effects were tested in the SK-BR-3 (R175H) and BT-549 (R249S) cells that contained p53 structural mutants and aggregates, or for negative control purposes, in MDA-MB-468 (R273H) containing p53 contact mutant and MCF-7 (WT) cells. Strikingly, IPI-504 treatment in SK-BR-3 (R175H) and BT-549 (R249S) cells promoted the mitochondrial translocation of apoptotic enablers Bax and PINK1, and the release of Cyto c, indicating that IPI-504 activated the mitochondrial apoptosis pathway in the presence of PTX or DOX (Figure 6A-B). Consequently, IPI-504 treatment increased cell apoptosis but reduced surviving colonies of SK-BR-3 (R175H) and BT-549 (R249S) cells in the presence of PTX or DOX, indicating that IPI-504 increased the sensitivity of chemotherapy (Figure S5A-B). Notably, IPI-504 showed no significant effects on cell apoptosis and chemotherapy efficacy in MDA-MB-468 (R273H) and MCF-7 (WT) cells in the presence of PTX or DOX (Figure 6A-B and Figure S5A-B). Furthermore, the therapeutic potential of IPI-504 was examined in the mouse tumor models with PTX treatment. Consistently, we found that intraperitoneal administration of IPI-504 substantially reduced the orthotopic tumor burden in mice transplanted with SK-BR-3 (R175H) and BT-549 (R249S) cells, while only minor repression of the growth of MDA-MB-468 (R273H) and MCF-7 (WT) tumors was observed, suggesting that IPI-504 rendered tumor cells with mutant p53 aggregates hypersensitive to chemotherapy (Figure 6C and Figure S5C). These results reveal that IPI-504 selectively increases the chemotherapy efficacy in breast cancer cells that express high-level mutant p53 aggregates.

To further determine whether IPI-504 promotes chemotherapy via suppression of mutant p53 aggregates, the p53-null 4T1 was transduced with BAG2 and trp53^R172H^ that formed mutant p53 aggregates, with BAG2 and trp53^R172H-I252R^ or shBAG2 and trp53^R172H^ that could not form aggregates. Notably, IPI-504 showed robust repression of the tumor growth of 4T1-BAG2-trp53^R172H^, but only slightly repressed the tumor growth of 4T1-BAG2-trp53^R172H-I252R^ and 4T1-shBAG2-trp53^R172H^ (Figure 6D and Figure S6A). IF analysis showed that IPI-504 remarkably diminished the aggregated mutant p53 protein in 4T1-BAG2-trp53^R172H^ tumors, while there was no p53 aggregation in 4T1-BAG2-trp53^R172H-I252R^ and 4T1-trp53^R172H^-shBAG2 tumor sections (Figure 6E and Figure S6B-C). Moreover, TUNEL assays of xenografts revealed that IPI-504 promoted cell apoptosis in 4T1-BAG2-trp53^R172H^ but not in 4T1-BAG2-trp53R^172H-I252R^ or 4T1-trp53^R172H^-shBAG2 xenografts (Figure 6F and Figure S6D-E). Importantly, IPI-504 effectively reduced lung metastasis and promoted apoptosis of 4T1-BAG2-trp53R^172H^ cells, but did not affect 4T1-BAG2-trp53^R172H-I252R^ and 4T1-trp53^R172H^-shBAG2, which did not form p53 aggregates (Figure 6G-H and Figure S6F-G). Consistently, IPI-504 selectively repressed the survival of 4T1-BAG2-trp53^R172H^ cells, but not that of 4T1-BAG2-trp53R^172H-I252R^ and 4T1-shBAG2-trp53R^172H^ cells after PTX treatment (Figure S6H). These results reveal that IPI-504 increases the sensitivity of chemotherapy via suppression of mutant p53 aggregates in breast cancer.

### Prognostic value of BAG2 and misfolded p53 expression

**Figure 7:**
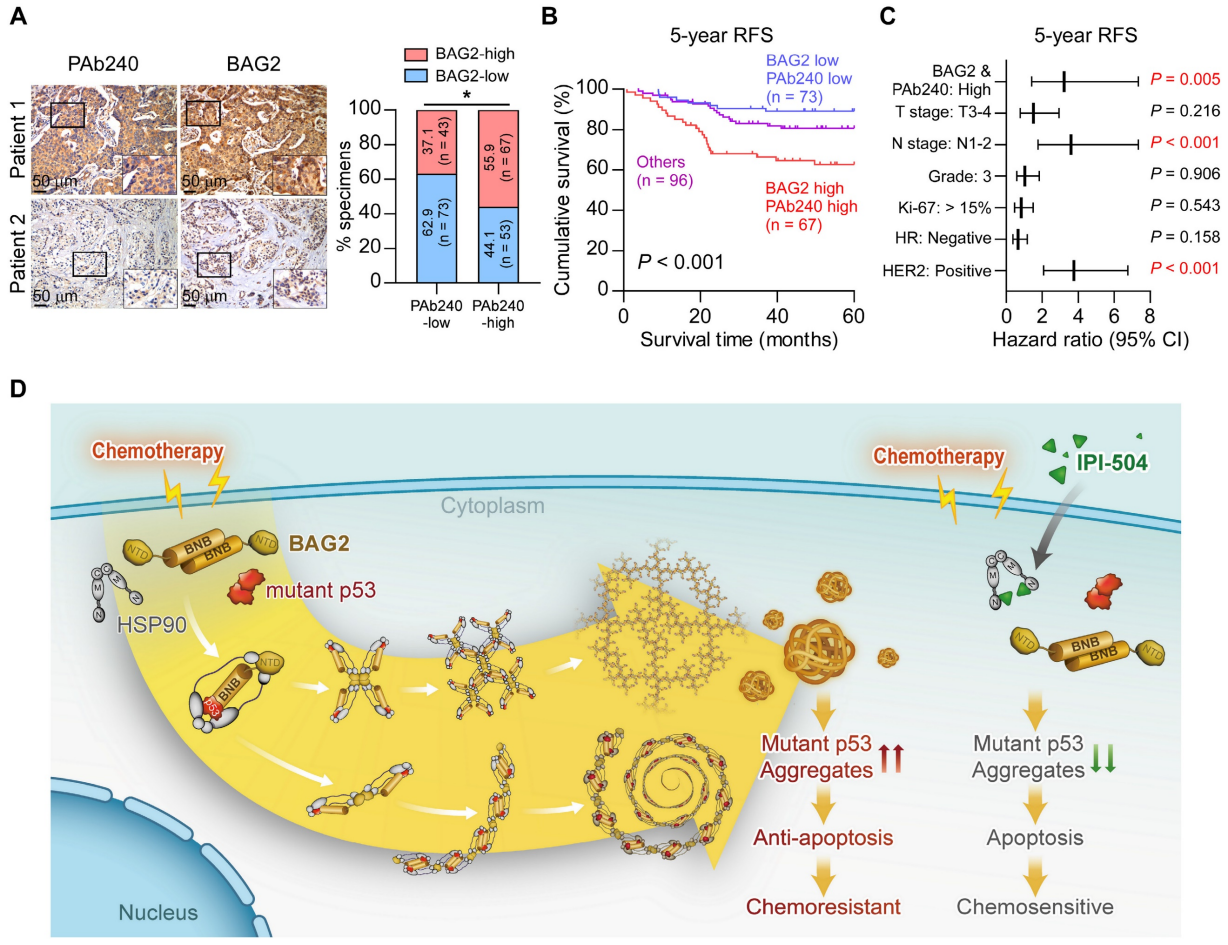
** Prognostic value of BAG2 and misfolded p53 expression. (A)** Representative images and correlation analysis of misfolded p53 (PAb240) and BAG2 in 236 breast cancer specimens. A two-sided χ^2^ test was used to evaluate the correlation. **(B)** The patient specimens were divided into three groups according to the BAG2 and misfolded p53 expression. Kaplan-Meier survival curves of 5-year RFS for these three groups (BAG2-low and PAb240-low, n = 73; BAG2-high and PAb240-high, n = 67; others, n = 96; *P* < 0.001, log-rank test).** (C)** Multivariate Cox regression analysis to evaluate the significance of the association between high BAG2/PAb240 signature and 5-year RFS in the presence of other clinical variables in breast cancer patients. **(D)** Proposed model. A schematic model depicting the regulation of exacerbated mutant p53 aggregate mediated by BAG2. See text for details. *P < 0.05.

Finally, we analyzed the clinical relevance and prognostic value of BAG2 and misfolded p53 expression in breast cancer. We found that BAG2 expression was significantly and positively correlated with misfolded p53 expression (Figure 7A). We further divided the specimens into three groups according to the status of BAG2 and misfolded p53 expression. Correlation analysis revealed that tumors with high BAG2 expression and misfolded p53 were significantly associated with aggressive features, including advanced T classification, lymph node metastasis, and relapse in breast cancer (Table S3). Moreover, breast cancer patients with a signature of high BAG2 and misfolded p53 expression had significantly poorer 5-year relapse-free survival (Figure 7B). In addition, the signature of high BAG2 and misfolded p53 expression could also be recognized as independent prognostic factors as suggested by the multivariate Cox regression analysis (Figure 7C and Table S4). These data indicate that breast cancer patients with BAG2-mutant p53 aggregates had a poorer prognosis. In summary, our findings suggest that BAG2 specifically binds to structural mutant p53 and promotes mutant p53 aggregation by recruiting HSP90, reducing the apoptosis of breast cancer cells to chemotherapeutic drugs, thereby conferring chemoresistance, and contributing to poor clinical outcomes in breast cancer (Figure 7D).

## Discussion

Chemoresistance is still a major challenge in the clinical management of breast cancer which induces tumor recurrence and correlates with poor prognosis. Identifying the pivotal factors that drive breast cancer chemoresistance and developing promising therapeutics for improving treatment outcomes are urgently needed. In the present study, we revealed that BAG2 was significantly upregulated in relapse breast cancer specimens and correlated with poor survival in patients with breast cancer. Further investigation indicated that BAG2 preferentially binds to structural mutant p53 and exacerbates mutant p53 aggregates by recruiting HSP90. Administration of IPI-504, an inhibitor of HSP90, selectively recovered the sensitivity of breast cancer cells with BAG2-induced mutant p53 aggregate to chemotherapeutic regimens. Our findings propose a novel mechanism for exacerbating mutant p53 aggregates and suggest that BAG2 is a potent prognostic biomarker and a promising therapeutic target against breast cancer chemoresistance.

Although the standard functions of the p53 protein are strongly associated with tumor suppression, mutant p53 and aggregates are involved in cancer progression 42. By connecting with other co-factors, mutant p53 may obtain the gain-of-function (GoF) subtype, including increased affinity to responsive elements, transcription factors, and other regulatory proteins 43, 44. Mutant p53 aggregates have been observed in a variety of tumors, and these conformations have oncogenic functions in cancer progression. Notably, p53 mutations are the most frequent genetic alterations in breast cancer, accounting for nearly 30% of breast cancer, with mutant p53 aggregate being observed in breast cancer patients' specimens 45. Despite the central role of mutant p53 aggregates in the hallmarks of breast cancer, mutant p53 status has not yet been used in the management of breast cancer 46. Therefore, targeting these oncogenic conformations may be a promising strategy for breast cancer treatment.

Several previous studies about BAG2 have indicated a significant role in cancer progression. For instance, LOXL1 and BAG2 proteins can interact in glioma cells, preventing BAG2-K186 ubiquitylation depending on LOXL1 enzymatic activity and stabilizing BAG2 to ultimately promote cell survival 18. In addition, BAG2 is deubiquitinated and stabilized by USP49, and knockdown of USP49 markedly inhibits colorectal cancer proliferation, colony formation, and chemoresistance resistance *in vitro* 19. BAG2 is associated with the progression and prognosis of hepatocellular carcinoma 20. BAG2 is related to poor prognosis and promotes the proliferation, invasion, and migration of oral squamous cell carcinoma cells by activating the MAPK signaling pathway 21. Recently, a study reported that BAG2 is a novel mutant p53 binding protein that promotes mutant p53 accumulation by attenuating MDM2-mediated ubiquitination degradation and is critical for mutant p53 GoF in tumorigenesis 22. Nevertheless, our research found out that BAG2 selectively interacts with structural mutant p53, but not with wild-type or contact mutant p53, and promotes mutant p53 aggregation by recruiting HSP90. Furthermore, we found that both BNB and CC motifs are necessary, but not sufficient, for the complete function of BAG2 in the formation of amyloid-like mutant p53 aggregates. Importantly, BAG2-induced exacerbation of mutant p53 aggregates contributes to chemoresistance in breast cancer. Therefore, we revealed a novel mechanism for BAG2 driving tumor malignancy and chemoresistance through promoting mutant p53 aggregates and counteracting adverse stimuli from chemotherapeutic regimens, which is independent of stabilizing mutant p53 protein, indicating that BAG2 has various effects on mediating p53 GoF and serves as an important factor in rendering chemoresistance in breast cancer.

HSP90 is an emerging target for cancer therapy because of its significant role in maintaining the activity and stability of key oncogenic signaling proteins 47. IPI-504, a novel, potent, and selective HSP90 inhibitor was designed to overcome the therapeutic limitations of earlier HSP90 inhibitors with potential advantages over those currently in development. The anti-cancer effects of IPI-504 have been demonstrated in multiple *in vitro* and *in vivo* models of cancer, leading to its clinical development in various phase II studies 48. Although there is a good preclinical rationale for the use of the IPI-504 in patients with carcinoma, only a few have shown clinical benefits. This might be due to the difficulty in identifying a precise clinical indication for the IPI-504. In the present study, HSP90 was found to be required for BAG2-mediated p53 aggregation. Furthermore, IPI-504 treatment selectively inhibited cell survival, tumor growth, and metastasis of cancer cells with aggregated mutant p53 following treatment with chemotherapeutic regimens. Therefore, mutant p53 aggregates may be considered a sensitive client substrate of HSP90, and targeting HSP90 might be a highly selective and efficient therapy against chemoresistance in breast cancer with mutant p53 aggregates.

In conclusion, our study demonstrated that BAG2 serves as an oncogene that promotes and exacerbates the mutant p53 aggregates by recruiting HSP90, and consequently contributes to chemoresistance in breast cancer. Importantly, the administration of IPI-504, an inhibitor of HSP90, selectively inhibited the proliferation and metastasis of breast cancer cells with BAG2-induced mutant p53 aggregates under chemotherapeutic regimens. Therefore, our study proposed a novel mechanism of breast cancer chemoresistance and suggested that BAG2 and mutant p53 aggregates might be potential clinical markers for identifying a subgroup of patients who are sensitive to IPI-504.

## Supplementary Material

Supplementary figures and tables.
