# Supplementary material for: BAG2 drives chemoresistance of breast cancer by exacerbating mutant p53 aggregate
Source: Theranostics. 2023 Jan 1;13(1):339–54. doi: 10.7150/thno.78492 (PMC9800719; doi:10.7150/thno.78492)
Supplement: Supplementary file 1 — Supplementary figures and tables. [file thnov13p0339s1.pdf]

Supplemental Data

Supplementary Figures

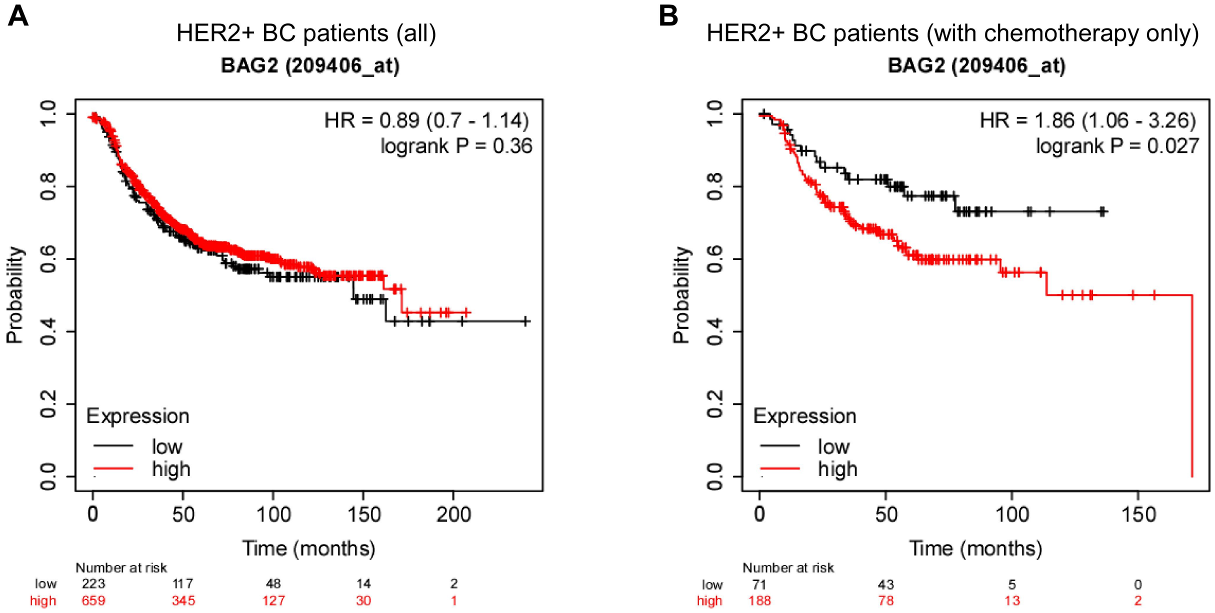

Figure S1. (A-B) Kaplan-Meier curves indicate the prognostic value of BAG2 expression in the RFS for HER2-positive breast cancer patients (A) or HER2-positive breast cancer patients treated with chemotherapy only (B) from online database (http://kmplot.com/analysis).

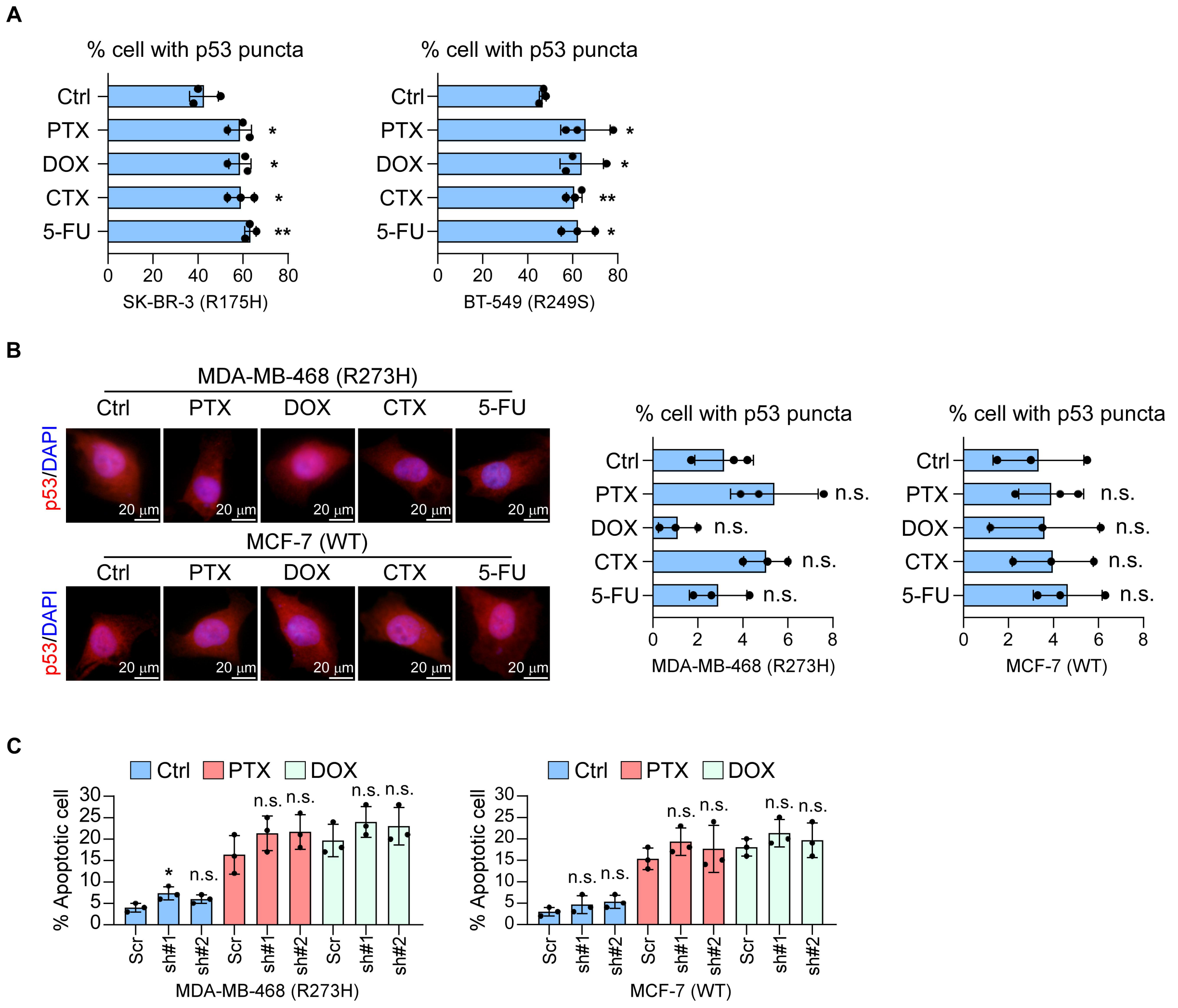

Figure S2. **(A)** Quantification of p53 aggregates in SK-BR-3 (R175H) and BT-549 (R249S) cells which harbor structural mutants under stationary, PTX, DOX, CTX, and 5-FU treatment. **(B)** IF staining of p53 (red) with anti-p53 DO-1. Shown are the representative images (left panel) and quantification (right panel) of endogenous aggregations of p53 proteins in MDA-MB-468 (R273H) cells, which harbor contact mutants and MCF-7 (WT) cells, under the indicated conditions. **(C)** Quantification of annexin V and PI staining for the MDA-MB-468 (R273H) and MCF-7 (WT) cells treated with PTX or DOX. Histograms show the portion of annexin V+ cells in each group. Each error bar represents the mean ± SD of three biological replicates. Two-sided Student's t test was used for all panels. *$P < 0.05$, **$P < 0.01$, and n.s.: not significant.

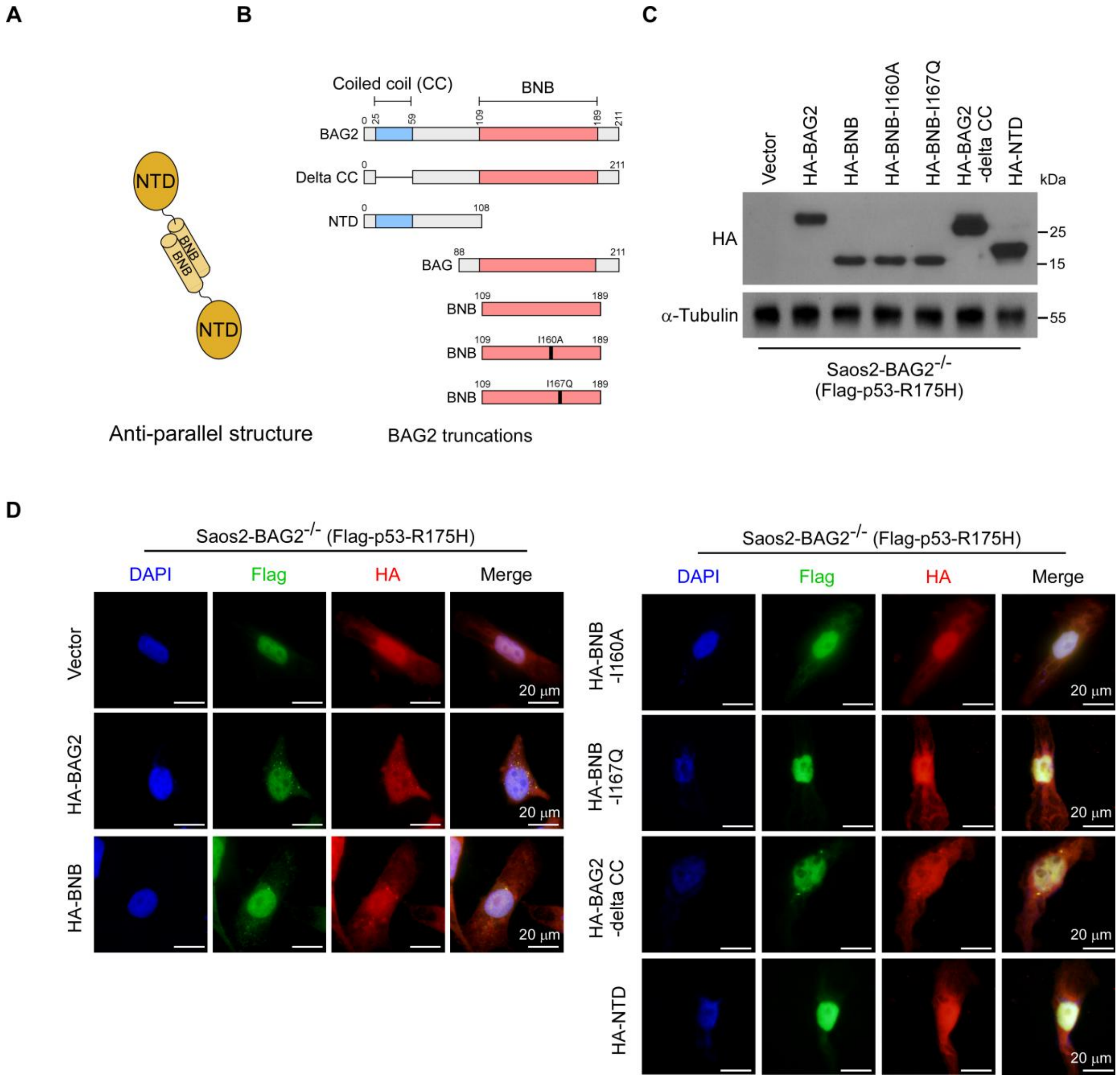

Figure S3. (A) Schematic illustration of the BNB and NTD structures and the antiparallel dimer structure of the BAG2 protein. **(B-C)** Schematic illustration and confirmation of the BAG2 truncation constructs. **(D)** Representative images of immunofluorescence (IF) staining of Flag and HA co-aggregations in Saos2-BAG2$^{-/-}$ cells transfected with the indicated truncations of BAG2.

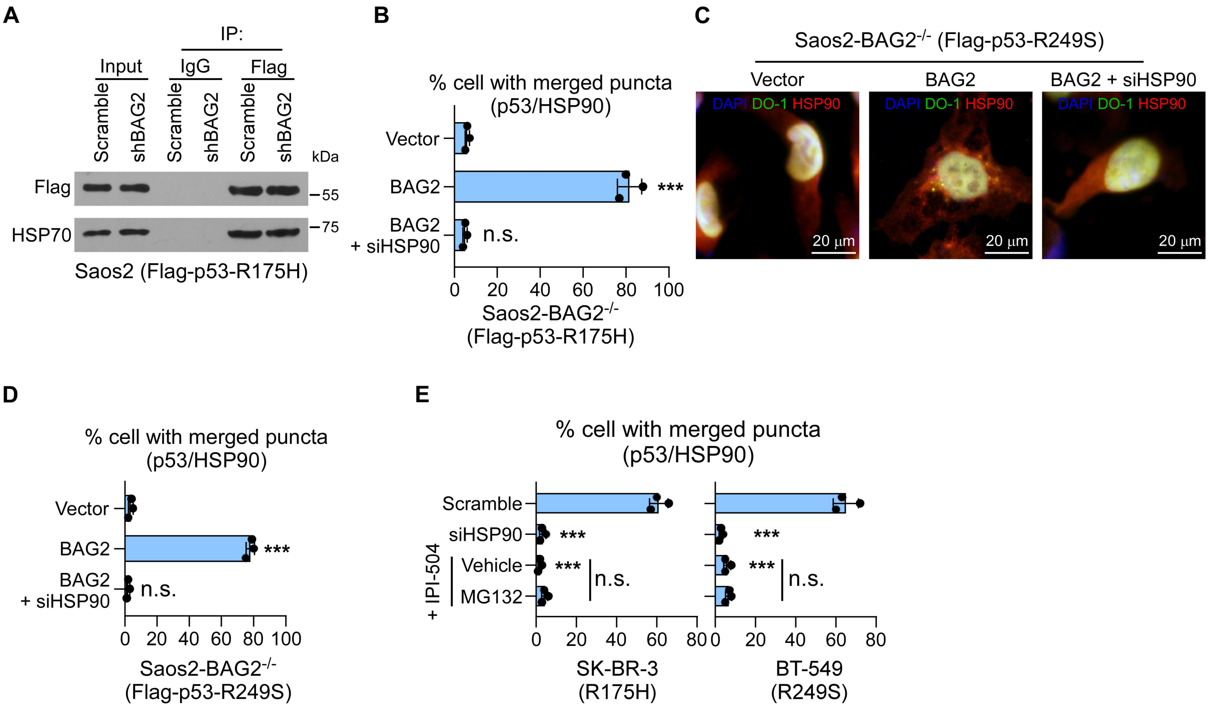

Figure S4. **(A)** Saos2-p53-R175H cells with silencing HSP90 were followed by IP assays and analyzed by western blotting. **(B)** Immunofluorescence (IF) staining quantification of p53-R175H and HSP90 aggregations in Saos2-BAG2$^{-/-}$ (Flag-p53-R175H) cells transfected with vector, BAG2, or BAG2 and HSP90 siRNA. **(C and D)** Immunofluorescence (IF) staining (C) and quantification (D) of p53-R249S and HSP90 aggregations in Saos2-BAG2$^{-/-}$ cells transfected with vector, BAG2, or BAG2 and HSP90 siRNA. **(E)** Quantification of p53/HSP90 aggregations in SK-BR-3 and BT-549 cells treating with or without IPI-504 and MG-132 (10 μM for 6 hours). Each error bar in B, D and E represents the mean ± SD of three biological replicates. Two-sided Student's t test was used for all panels. ****P* < 0.001, n.s.: not significant.

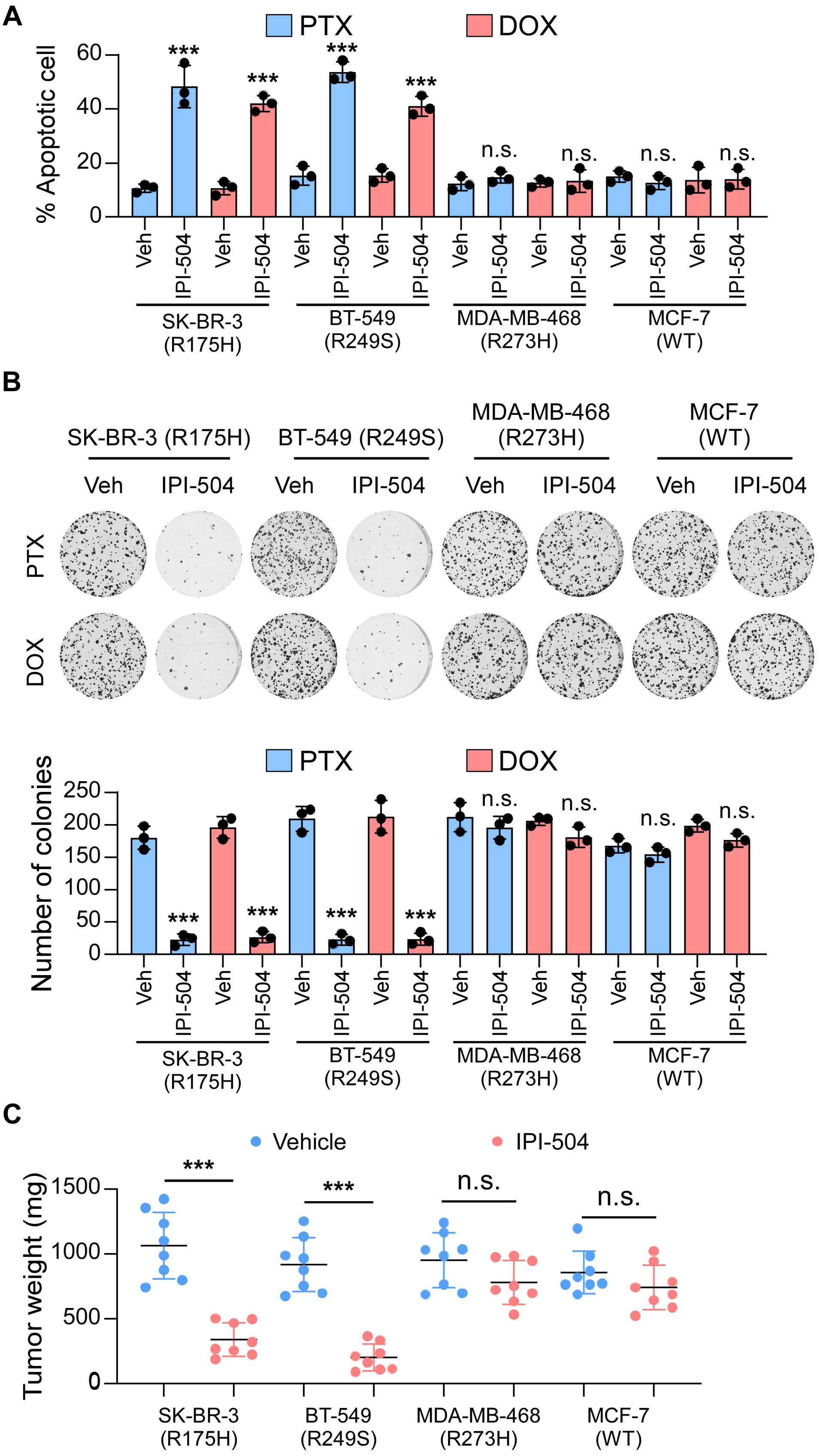

Figure S5. (A and B) The number of surviving colonies and apoptotic percentage of the SK-BR-3, BT-549, MDA-MB-468 and MCF-7 cells with or without treatment of IPI-504. **(C)** The tumor

weights of the indicated cells with or without treatment of IPI-504. Each error bar in A and B represents the mean ± SD of three biological replicates. Error bar in C represents the mean ± SD derived from tumor mouse models (n = 8 mice/group). Two-sided Student's t test was used for all panels. ***P < 0.01, n.s.: not significant.

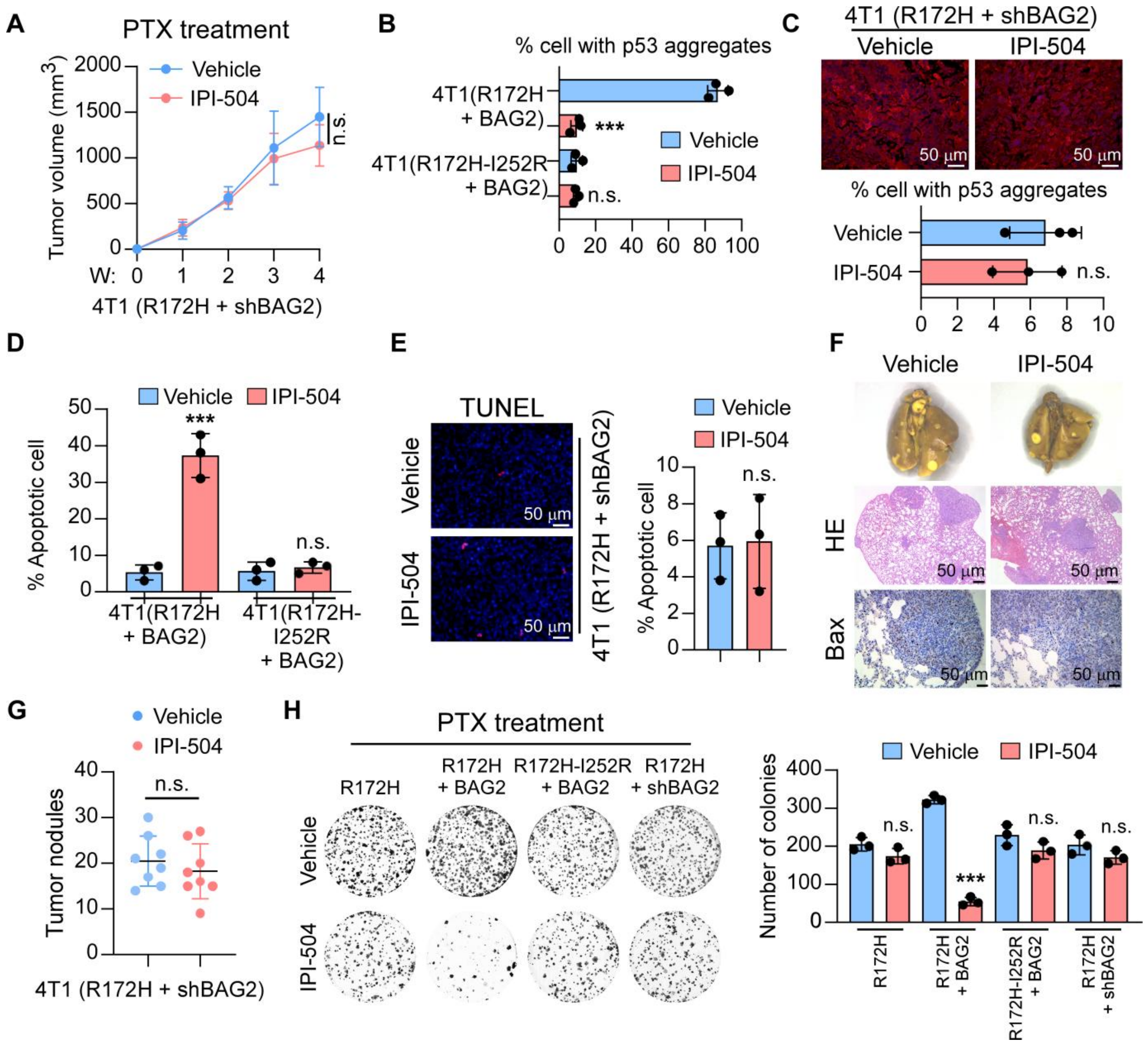

Figure S6. (A) The tumor volume of 4T1 (R172H + shBAG2) xenograft with or without IPI-504 treatment. **(B)** Quantification of aggregated p53 in 4T1 (R172H + BAG2) and 4T1 (R172H-I252R + BAG2) xenografts treating with or without IPI-504 were shown. **(C)** The representative images and quantification of aggregated p53 in the indicated sections xenografts were examined by IF staining. **(D)** Apoptosis index were calculated using the proportions of TUNEL positive cells in the indicated xenograft sections. **(E)** TUNEL staining and apoptosis index in the 4T1 (R172H + shBAG2) orthotopic tumors. **(F)** Images, HE staining and IHC of Bax staining of lung metastases. **(G)** Quantification of visible surface metastatic lesions in the indicated tumors. **(H)** Colony formation assays of the indicated cells with or without IPI-504 treatments. Surviving colonies were counted. Each error bar in A and G represents the mean ± SD derived from tumor mouse models (n = 8 mice/group). Each error bar in B, C, D, E and H represents the mean ± SD of three biological

replicates. Two-sided Student's t test (B, C, D, E, G, H), or One-way repeated-measures ANOVA test (A) was used for statistical analysis. ***P < 0.001, n.s.: not significant.

Supplementary Tables

Table S1, Clinicopathological characteristics of 236 breast cancer patients

Parameters	Number of cases (%)
**Gender**	
Female	236 (100)
Male	0 (0)
**Age**	
≤ 49	128 (54.2)
> 49	108 (45.8)
**Clinical stage**	
I-II	154 (65.3)
III-IV	82 (34.7)
**T classification**	
T1-2	203 (86.0)
T3-4	33 (14.0)
**N classification**	
N0	111 (47.0)
N1-3	125 (53.0)
**Histologic grade**	
G1-2	139 (58.9)
G3	97 (41.1)
**Hormone receptor**	
Negative	109 (46.2)
Positive	127 (53.8)
**HER2 status**	
Negative	154 (65.3)
Positive	82 (34.7)
**Ki67**	
≤ 15%	112 (47.5)
> 15%	124 (52.5)
**TNBC**	
No	165 (69.9)
Yes	71 (30.1)
**Relapse**	
No	160 (67.8)
Yes	76 (32.2)

**BAG2 expression**	
Low	126 (53.4)
High	110 (46.6)
**PAb240**	
Low	116 (49.2)
High	120 (50.8)
**BAG2 combines with PAb240**	
High/high	67 (28.4)
High/low or low/high	96 (40.7)
Low/low	73 (30.9)

Table S2, Correlation between BAG2 and clinicopathological characteristics of 236 breast cancer patients

	BAG2		
Characteristics	low	high	*P* values
**Age**			
≤ 49	72	56	0.338
> 49	54	54	
**Clinical stage**			
I-II	88	69	0.248
III-IV	38	41	
**T classification**			
T1-2	113	90	0.082
T3-4	13	20	
**N classification**			
N0	64	47	0.216
N1-3	62	64	
**Histologic grade**			
G1-2	77	62	0.460
G3	49	48	
**Hormone receptor**			
Negative	56	53	0.566
Positive	70	57	
**HER2 status**			
Negative	86	68	0.300
Positive	40	42	
**Ki67**			
≤ 15	60	52	0.958
> 15	66	58	
**TNBC**			
No	89	76	0.796
Yes	37	34	
**Relapse**			
No	101	59	0.001
Yes	25	51	

Table S3, Correlation between BAG2 combined with PAb240 and clinicopathological characteristics of 236 breast cancer patients

	BAG2/PAb240			
Characteristics	-/-	+/-	+/+	*P* values
**Age**				
≤ 49	41	57	30	0.170
> 49	32	39	37	
**Clinical stage**				
I-II	53	64	37	0.091
III-IV	20	32	30	
**T classification**				
T1-2	67	82	54	0.159
T3-4	6	14	13	
**N classification**				
N0	43	43	25	0.032
N1-3	30	53	42	
**Histologic grade**				
G1-2	41	59	39	0.779
G3	32	37	28	
**Hormone receptor**				
Negative	30	49	30	0.422
Positive	43	47	37	
**HER2 status**				
Negative	49	63	42	0.855
Positive	24	33	25	
**Ki67**				
≤ 15	34	44	34	0.813
> 15	39	52	33	
**TNBC**				
No	53	64	48	0.661
Yes	20	32	19	
**Relapse**				
No	59	66	35	0.001
Yes	14	30	32	

Table S4, Univariate and multivariate analysis of factors associated with 5-year relapse-free survival in 236 breast cancer patients

Characteristics	Univariate analysis		Multivariate analysis	
	HR (95% CI)	*P* values	HR (95% CI)	*P* values
**BAG2 combines with PAb240** (high/high)	2.082 (1.415-3.063)	< 0.001	3.219 (1.413- 7.337)	0.005
**T classification** (T3-4)	2.622 (1.392-4.938)	0.003	1.518 (0.784- 2.938)	0.216
**N classification** (N1-3)	3.858 (1.974-7.539)	< 0.001	3.605 (1.771- 7.336)	< 0.001
**HR status** (negative)	0.844 (0.485-1.469)	0.549	0.660 (0.371- 1.174)	0.158
**HER2 status** (positive)	3.291 (1.867-5.799)	0.001	3.756 (2.088- 6.755)	< 0.001
**Ki67** (> 15%)	1.069 (0.613-1.864)	0.814	0.834 (0.464- 1.499)	0.543
**Histologic grade** (G3)	1.317 (0.756-2.294)	0.331	1.035 (0.585- 1.832)	0.906

HR, hazard ratio; CI, confidence interval.
